# Artificial intelligence in forensic science: a systematic review. Part II: long-range postmortem interval estimation

**DOI:** 10.1007/s00414-026-03856-4

**Published:** 2026-06-12

**Authors:** Valentina Bugelli, Francesco Calabrò, Jessika Camatti, Rossana Cecchi, Marco Di Paolo, Lorenzo Franceschetti

**Affiliations:** 1 University of Parma, Parma, Italy; 2 University of Modena and Reggio Emilia, Modena, Italy; 3 University of Pisa, Pisa, Italy; 4 University of Milano, Milan, Italy

**Keywords:** Artificial intelligence, Machine learning, Postmortem interval, Forensic pathology, Decomposition, Forensic imaging

## Abstract

Postmortem interval (PMI) estimation remains a major challenge in forensic medicine due to the complex and multifactorial nature of decomposition processes. In recent years, artificial intelligence (AI) and machine learning techniques have been increasingly applied to improve PMI prediction. This systematic review aimed to evaluate the current evidence on AI-based models developed for PMI estimation. A systematic literature search was conducted in PubMed/MEDLINE and Scopus from database inception to 1 March 2026, following PRISMA 2020 guidelines. Studies were included if they applied AI, machine learning, or deep learning methods to estimate PMI using real postmortem datasets. Data extraction included study characteristics, data modality, AI model architecture, validation strategy, and reported performance metrics. A total of 64 studies met the inclusion criteria. The most common approach involved microbiome-based models (*n* = 29), followed by metabolomics and proteomics approaches (*n* = 11) and imaging-based AI models (*n* = 11). Random Forest algorithms were the most frequently used machine learning method, particularly in microbiome studies. Reported predictive performance varied widely across studies, with several models achieving high accuracy or low prediction errors depending on the data modality and PMI range investigated. AI represents a promising tool for improving the accuracy and objectivity of PMI estimation by enabling the integration of complex forensic datasets. However, current evidence is limited by heterogeneous methodologies, small datasets, and a lack of external validation. Future research should focus on large multicenter datasets, standardized validation protocols, and multimodal AI models integrating diverse forensic data sources.

**Supplementary Information:**

The online version contains supplementary material available at 10.1007/s00414-026-03856-4.

## Introduction

The estimation of the postmortem interval (PMI), defined as the time elapsed since death, represents one of the most critical tasks in forensic investigations. Accurate determination of PMI can assist in reconstructing the sequence of events surrounding death, narrowing investigative timelines, and corroborating or challenging witness testimonies and alibis. Over the past decades, numerous biological, biochemical, and environmental indicators have been investigated for this purpose. However, despite substantial research efforts, PMI estimation remains a complex challenge due to the multifactorial nature of postmortem processes and the strong influence of environmental conditions on decomposition dynamics [1, 2].

Traditional approaches to PMI estimation are often based on empirical observations or single biological indicators. These methods can be substantially influenced by external factors. Consequently, classical techniques frequently provide relatively broad time intervals rather than precise estimates, particularly in advanced stages of decomposition. In addition, interpretation of postmortem findings often relies on expert judgment, which may introduce inter-observer variability. These limitations have prompted the search for more objective and reproducible analytical methods capable of integrating multiple sources of biological and environmental information to improve the accuracy of PMI estimation [3–5].

Artificial intelligence (AI), encompassing machine learning (ML) and deep learning techniques, has experienced rapid development across biomedical and forensic sciences. These computational approaches are capable of identifying complex and non-linear relationships within large datasets and integrating heterogeneous variables that are difficult to analyze using conventional statistical methods. In forensic medicine, AI applications have progressively expanded to areas such as forensic imaging interpretation, injury pattern analysis, identification of human remains, and predictive modelling of biological parameters relevant to death investigations [6, 7].

More recently, AI-based models have been proposed for the estimation of PMI using a wide variety of data sources, including postmortem imaging, biochemical and metabolomic markers, microbial succession, environmental parameters, and forensic entomology datasets. These approaches aim to enhance predictive accuracy by capturing complex interactions among multiple factors involved in decomposition processes. Early studies have reported promising results [8, 9], suggesting that AI-driven models may outperform traditional approaches, particularly when applied to large multidimensional datasets.

Despite the increasing number of studies investigating AI-based approaches for PMI estimation, the available literature remains highly heterogeneous with respect to study design, data sources, modelling strategies, and validation frameworks. Differences in dataset size, environmental variability, and performance metrics make it challenging to compare findings across studies and to assess the real forensic applicability of these models [7]. Moreover, many studies rely on small experimental datasets or animal models, limiting the generalizability of the results to real forensic casework.

The aim of the present systematic review is to evaluate the current evidence on artificial intelligence–based models developed for postmortem interval estimation. Specifically, this review aims to examine the methodological approaches adopted in AI-driven PMI prediction, the types of data employed, model validation strategies, and the reported predictive performance. By synthesizing the available literature, this work seeks to provide a comprehensive overview of the current state of the field and to identify potential directions for future research and forensic application.

## Materials and methods

### Study design and research question

This study was designed as a systematic review aimed at evaluating artificial intelligence (AI) applications in postmortem interval (PMI) estimation. The review was conducted according to the Preferred Reporting Items for Systematic Reviews and Meta-Analyses (PRISMA 2020) guidelines, ensuring transparency and reproducibility in study identification, selection, and synthesis [10]. The study protocol was prospectively registered on the Open Science Framework (OSF) to ensure methodological transparency and reproducibility. A publicly accessible view-only version of the protocol is available at: https://osf.io/j3ymz/overview?view_only=f0e11d87cbb14529833330e94ca762e5.

The objective of this review was to investigate the performance, methodological approaches, and forensic applicability of AI-based models developed for estimating the postmortem interval using biological, imaging, biochemical, entomological, or environmental data.

### Eligibility criteria

Studies were included if they met the following criteria: original research articles applying AI, machine learning, or deep learning techniques; studies addressing postmortem interval estimation or time-since-death prediction; use of forensic or experimentally simulated postmortem datasets; quantitative evaluation of model performance (e.g., MAE, RMSE, accuracy, correlation coefficients); peer-reviewed publications written in English. Studies involving both human postmortem data and experimentally generated animal models used to simulate decomposition were considered eligible.

Exclusion criteria were: studies using purely statistical or regression-based approaches without AI or machine learning methods; narrative reviews, editorials, conference abstracts without full data, and methodological commentaries; studies focused exclusively on decomposition description without predictive modelling; articles lacking objective performance metrics.

### Information sources and search strategy

MEDLINE (via PubMed) and Scopus were searched from database inception to the final search date (1 March 2026), supplemented by backward and forward citation tracking. Database-specific strategies are provided in Supplementary Appendix 1. Manual screening of reference lists from eligible studies was performed to identify additional relevant publications. After duplicate removal, studies were screened independently by two reviewers through: title and abstract screening; full-text evaluation. Disagreements were resolved through consensus discussion.

### Data extraction

Data extraction was conducted using a standardized form developed prior to study selection. The following variables were collected: author(s), year, and journal; study design and forensic context; type of data used for PMI estimation (imaging, biochemical, molecular, entomological, environmental, multimodal); sample characteristics (human, animal models, experimental conditions); AI model architecture and training strategy; validation approach (internal or external validation); target outcome (continuous PMI estimation or interval classification); performance metrics (MAE, RMSE, correlation, accuracy); comparator methods (traditional PMI estimation approaches); reported limitations and sources of bias. Data extraction was performed independently by two reviewers and verified for consistency.

### Risk of bias and methodological quality assessment

Risk of bias was assessed using tools adapted to AI-based prediction studies, primarily based on the PROBAST framework for predictive modelling studies and QUADAS-2 principles when diagnostic accuracy designs were applicable. Because no standardized risk-of-bias tool currently exists specifically for artificial intelligence–based forensic PMI prediction studies, the assessment criteria were adapted according to methodological characteristics relevant to forensic AI applications.

The evaluation focused on: (i) dataset representativeness; (ii) consideration of environmental variability; (iii) validation strategy (internal versus external validation); (iv) potential risk of overfitting; (v) transparency and reproducibility of AI pipelines. Studies relying exclusively on small experimental datasets, animal-only decomposition models, or internal validation strategies were considered at increased risk of bias regarding generalizability and predictive robustness.

Each study was classified as presenting low, moderate, or high overall risk of bias. A detailed study-level assessment is provided in Supplementary Table S1.

## Results

### Study selection

The literature search identified a total of 269 records, including 148 records from PubMed/MEDLINE and 121 records from Scopus. After removing 80 duplicate records and one retracted article, 188 records remained for title and abstract screening. Of these, 118 records were excluded because they did not meet the eligibility criteria. The remaining 70 reports were assessed for full-text eligibility, of which two could not be retrieved.

**Fig. 1:**
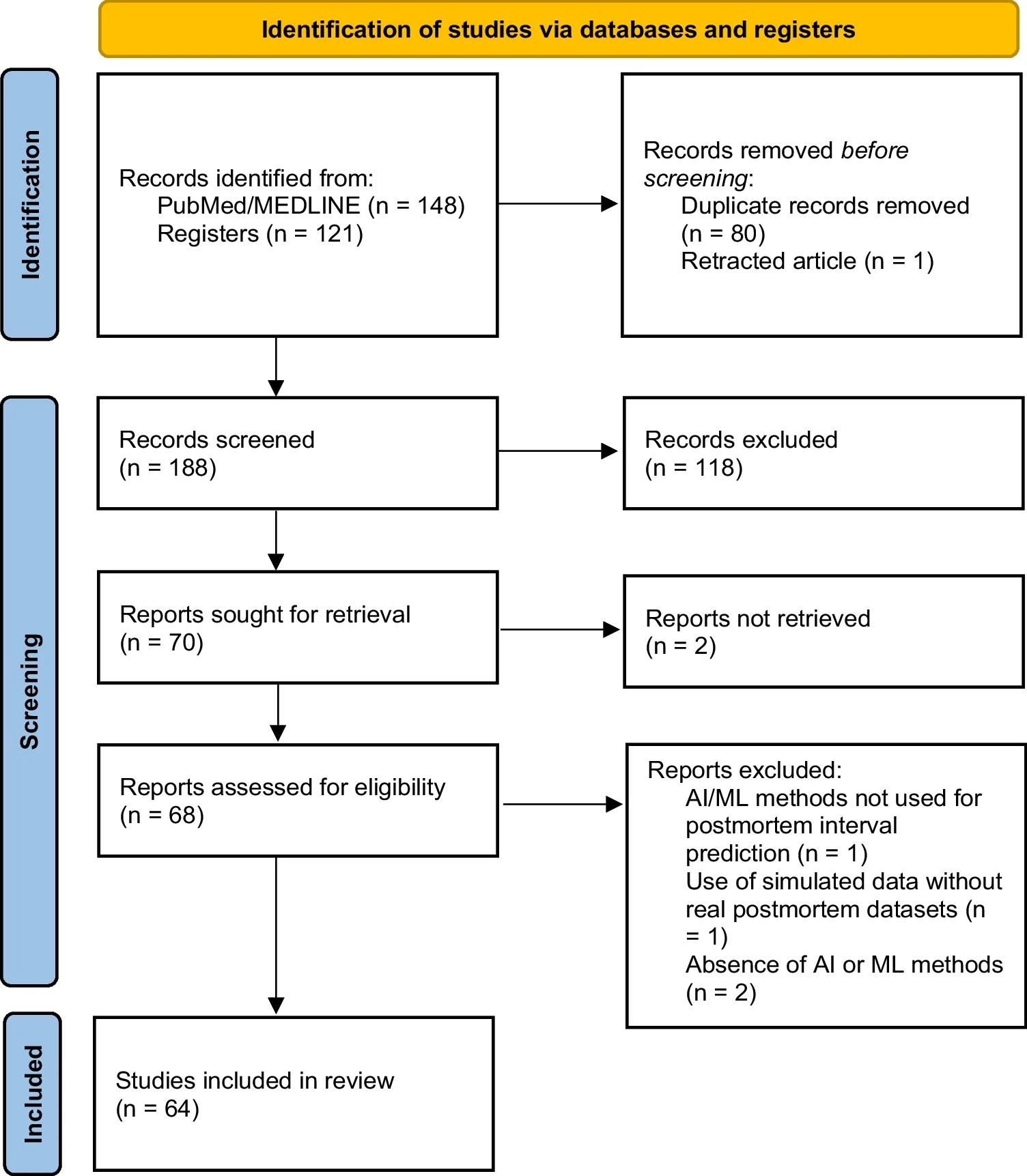
PRISMA Flow diagram of the systematic review

Following full-text evaluation, four additional studies were excluded due to the absence of artificial intelligence or machine learning approaches, the use of simulated datasets without real postmortem data, or the lack of predictive modelling of the postmortem interval. Ultimately, 64 studies met the inclusion criteria and were included in the qualitative synthesis [11–74]. The study selection process is illustrated in the PRISMA flow diagram (Fig. 1).

### Characteristics of included studies

The main characteristics of the included studies are summarized in Supplementary Table S2. Overall, the 64 studies investigated a broad range of biological, molecular, imaging, and environmental datasets for artificial intelligence–based PMI estimation. Most investigations relied on experimental animal decomposition models, particularly rodents and pigs, although several studies employed human cadavers, skeletal remains, or retrospective forensic datasets.

The included studies evaluated both continuous PMI prediction and classification-based approaches. Investigated PMI windows ranged from early postmortem changes occurring within hours after death to long-term decomposition intervals extending over several months or years. The principal methodological categories identified in the literature are summarized in Table 1 and discussed in detail in the following sections.

**Table 1:** Overview of artificial intelligence approaches for postmortem interval estimation in the studies included in this review (*n* = 64). Two studies were classified into more than one methodological category; therefore, the total number of category entries (*n* = 66) exceeds the total number of included studies (*n* = 64)

AI approach	Biological source	Common AI/ML models	Number of studies	Typical PMI window	Reported performance
Microbiome-based models	Skin, gut, oral cavity, soil, bone, burial environments	Random Forest, XGBoost, Gradient Boosting, ANN	29	Hours → months	MAE ≈ 0.6 h – 5 days; R^2^ up to 0.95
Metabolomics/proteomics	Blood, muscle, liver, multi-organ tissues	SVR, neural networks, Random Forest, stacking ensembles	11	Hours → days	MAE ≈ 0.07–2 days; AUC up to 0.98
Spectroscopy-based approaches	Vitreous humor, skeletal muscle, bone, puparia	FTIR, ATR-FTIR, NIR + SVR/ANN/PLS models	7	Hours → months	RMSE ≈ 1–2 days (early PMI); classification accuracy up to 100%
Imaging-based AI (radiomics/pathology)	PMCT scans, histopathology slides, corneal imaging, hyperspectral imaging	CNN, ResNet, Vision Transformer, radiomics ML classifiers	11	Early PMI (hours–days)	Accuracy ≈ 78–96%; AUC ≈ 0.75–0.96
Forensic entomology models	Blowfly larvae, pupae, puparia	ANN, SVR, XGBoost regression	6	Days → weeks	Accuracy ≈ 80–94%; MAE ≈ 1–7 developmental units
Environmental/taphonomic ML datasets	Forensic decomposition databases, GIS environmental variables	Random Forest, machine learning classifiers	2	Days → months	R^2^ ≈ 0.82

Abbreviations: *AI* Artificial Intelligence; *ANN* Artificial Neural Network; *AUC* Area Under the Receiver Operating Characteristic Curve; *CNN* Convolutional Neural Network; *FTIR* Fourier Transform Infrared Spectroscopy; *GIS* Geographic Information System; *MAE* Mean Absolute Error; *ML* Machine Learning; *NIR* Near-Infrared Spectroscopy; *PMCT* Postmortem Computed Tomography; *PMI* Postmortem Interval; *PMImin* Minimum Postmortem Interval; *PLS* Partial Least Squares; *R*^2^ Coefficient of Determination; *RF* Random Forest; *RMSE* Root Mean Square Error; *SVR* Support Vector Regression; *XGBoost* eXtreme Gradient Boosting; ATR-FTIR = Attenuated Total Reflectance Fourier Transform Infrared Spectroscopy

### Microbiome-based models

Microbiome-based approaches represented the largest group among the included studies, accounting for 29 of the 64 investigations. These models analyzed microbial succession in different anatomical and environmental contexts, including skin, gut, oral cavity, gravesoil, bone, burial environments, and aquatic decomposition settings.

Most studies employed Random Forest algorithms because of their ability to manage high-dimensional sequencing datasets and nonlinear biological interactions. Several investigations used murine or porcine experimental decomposition models, whereas others analyzed human cadavers or skeletal remains.

Human cadaver studies demonstrated promising predictive performance. Hu et al. analyzed intestinal microbiota from 63 cadavers and reported high predictive accuracy for PMI estimation [28], while Chen et al. investigated oral and nasal microbiota in 88 human swab samples and achieved mean absolute errors ranging from 2.16 to 5.14 days [18]. Other studies focused on environmental microbial communities associated with decomposition, including gravesoil bacteria, aquatic biofilms, and bone microbial succession.

Among experimental animal models, Liu et al. reported MAE values of approximately 20 h with coefficients of determination approaching R^2^ ≈ 0.95 in murine decomposition models [43]. Similarly, Zhao et al. demonstrated strong predictive performance using oral microbiome sequencing during rat decomposition, with R^2^ values approaching 0.94 [74].

Overall, microbiome-based models demonstrated some of the most consistent predictive performances across broad PMI ranges, although substantial variability remained related to environmental conditions, sample origin, and decomposition context.

### Metabolomics and proteomics approaches

A total of 11 studies investigated metabolomics- and proteomics-based approaches for PMI estimation. These methods aimed to identify biochemical alterations associated with postmortem degradation processes, including metabolite accumulation, tissue biochemical changes, and protein fragmentation.

Most investigations analyzed blood, skeletal muscle, liver tissue, or multi-organ datasets using mass spectrometry–based analytical platforms combined with machine learning algorithms such as support vector regression, Random Forest, neural networks, and ensemble learning models.

Several studies demonstrated particularly promising performance for early PMI estimation. Magnusson et al. applied neural network models to large-scale human blood metabolomic datasets including more than 4,800 individuals and reported mean absolute errors of approximately 1.45 days [47]. Similarly, Li et al. developed a multi-omics stacking ensemble model integrating metabolomics, proteomics, and FTIR data, achieving AUC values up to 0.98 with very low prediction errors [38]. Fan et al. further demonstrated robust predictive performance across multiple temperature conditions using multi-organ GC–MS metabolomics datasets [25].

Proteomic approaches also showed encouraging results. Du et al. combined multi-organ proteomic fragments with ensemble machine learning methods and reported classification accuracies exceeding 0.89 in external validation cohorts [24]. Other studies investigated skeletal muscle or bone protein degradation patterns for late PMI estimation and demonstrated promising classification performance for long-term decomposition intervals.

Overall, metabolomics and proteomics approaches appeared particularly effective for early PMI estimation and multidimensional forensic datasets.

### Imaging-based AI models

Imaging-based artificial intelligence approaches were reported in 11 studies and included postmortem computed tomography (PMCT), pathology whole-slide imaging, corneal opacity analysis, and hyperspectral imaging techniques.

Recent investigations increasingly employed deep learning architectures such as convolutional neural networks (CNNs), ResNet models, DenseNet, and Vision Transformers. Imaging-based models were primarily applied to early PMI estimation and interval classification tasks.

Ivanov et al. developed a 3D convolutional neural network based on PMCT imaging datasets obtained from 123 human cadavers and demonstrated promising classification performance for different PMI intervals [30]. Klontzas et al. used PMCT radiomics combined with XGBoost classifiers and reported AUC values of approximately 0.75 for distinguishing PMI intervals below and above 12 h [33].

Histopathology whole-slide imaging represented another emerging field. An et al. and Liang et al. applied deep learning architectures to pathology imaging datasets derived from human and animal decomposition models, achieving classification accuracies ranging from approximately 78% to over 93% [11, 12, 41, 42].

Additional imaging-based approaches included corneal opacity analysis and hyperspectral imaging of entomological specimens. Overall, imaging-based AI demonstrated promising performance, particularly in controlled experimental settings and early PMI estimation scenarios.

### Spectroscopy-based approaches

Seven studies investigated spectroscopy-based approaches for PMI estimation, primarily using Fourier transform infrared spectroscopy (FTIR), attenuated total reflectance FTIR (ATR-FTIR), and near-infrared spectroscopy (NIR).

These methods analyzed vitreous humor, skeletal muscle, bone tissue, and insect puparia to detect biochemical alterations associated with decomposition. Artificial neural networks, support vector regression, partial least squares models, and Random Forest algorithms were commonly employed.

Several studies demonstrated excellent predictive performance during early PMI intervals. Zhang et al. reported ANN-based prediction errors of approximately 2 h using vitreous humor FTIR analysis [66], whereas Deng et al. achieved RMSE values of approximately 1.8 days using ATR-FTIR spectroscopy of human skeletal muscle [22]. Schmidt et al. also reported classification accuracies approaching 100% using NIR spectroscopy of human skeletal remains [53].

Other investigations explored long-term PMI estimation using weathered insect puparia or skeletal remains, suggesting that spectroscopy-based approaches may also provide value for extended decomposition intervals.

### Forensic entomology and environmental/taphonomic models

Six studies focused on forensic entomology–based AI models, primarily analyzing blowfly larvae, pupae, puparia, or cuticular hydrocarbons for minimum PMI estimation. Artificial neural networks, support vector regression, and XGBoost regression models were commonly applied to insect developmental datasets.

These studies generally demonstrated high predictive performance, with classification accuracies ranging from approximately 80% to 94%. Several investigations also reported promising applications of hyperspectral imaging and ATR-FTIR spectroscopy for estimating developmental stages of forensically important insects.

Environmental and taphonomic machine learning datasets were less frequently investigated. Weisensee et al. developed a large forensic taphonomy database integrating GIS environmental variables and decomposition features from more than 2,500 forensic cases, achieving R^2^ values of approximately 0.82 [61]. Similarly, Korgesaar et al. demonstrated reliable PMI interval classification using environmental and taphonomic variables derived from retrospective forensic datasets [34]. Sharif et al. and Yu et al. further demonstrated promising performance of machine learning and deep learning models applied to puparial chemical and morphological analysis for PMImin estimation [54, 64].

### Validation strategies and predictive performance

A wide range of machine learning algorithms were applied across the included studies. Random Forest represented the most frequently used approach, particularly in microbiome-based investigations, whereas deep learning architectures predominated in imaging-based studies.

Most studies relied on internal validation approaches, including cross-validation or train–test splits, while independent external validation cohorts were reported less frequently. Overall, AI-based models demonstrated promising predictive performance across different methodological categories, with reported MAE values ranging from a few hours to several days depending on the investigated PMI interval and biological matrix.

However, predictive performance varied substantially according to dataset size, environmental variability, decomposition conditions, and validation strategy. Studies employing external validation generally reported lower but more methodologically robust predictive performance compared with internally validated experimental datasets.

### Risk of bias assessment

The risk-of-bias assessment demonstrated substantial methodological heterogeneity across the included studies. Most studies were classified as presenting moderate risk of bias, mainly due to limited external validation, relatively small experimental datasets, and the frequent use of animal decomposition models.

Only a minority of studies employed independent external validation cohorts, whereas most investigations relied on internal validation approaches such as cross-validation or train–test splits, potentially leading to optimistic estimates of predictive performance.

Studies based on human forensic datasets combined with external validation generally demonstrated lower methodological risk and greater generalizability. Conversely, studies using highly controlled experimental conditions or limited sample sizes showed increased concerns regarding reproducibility and real-world forensic applicability.

The detailed study-level assessment is reported in Supplementary Table S1.

**Fig. 2:**
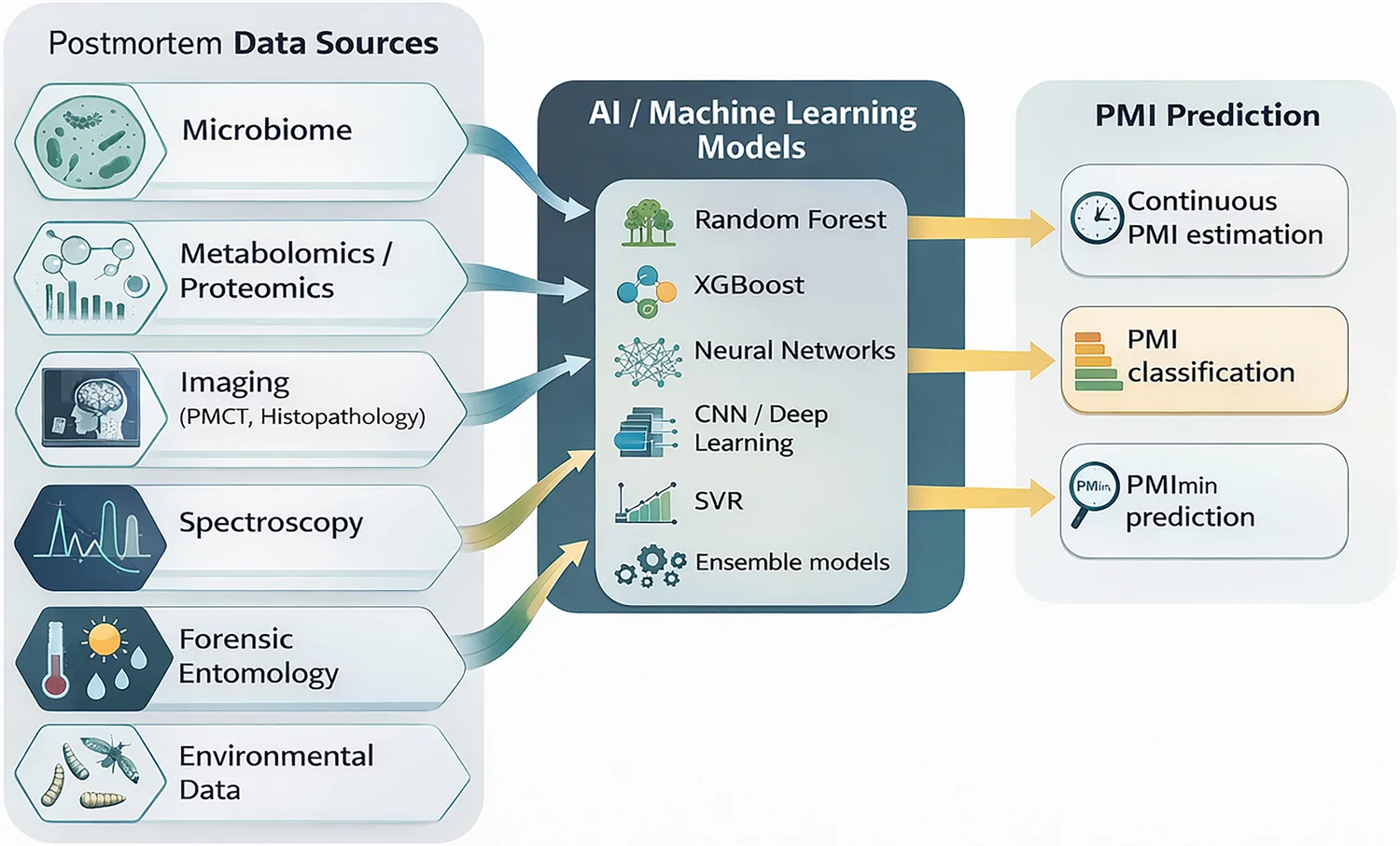
Conceptual framework of artificial intelligence–based approaches for postmortem interval estimation. Different types of postmortem data—including microbiome profiles, molecular biomarkers, imaging features, spectroscopic signals, entomological evidence, and environmental variables—can be integrated using machine learning and deep learning models to generate predictive estimates of the postmortem interval

Figure 2 illustrates the conceptual framework of AI-based approaches for postmortem interval estimation and the main categories of data sources and machine learning models identified in the literature.

Taken together, these findings suggest that AI-based PMI estimation models demonstrate substantial potential across multiple forensic domains, although methodological heterogeneity and limited external validation remain important limitations.

## Discussion

In recent years, AI and machine learning techniques have been increasingly applied in forensic research as tools capable of integrating complex datasets and identifying patterns that may improve PMI estimation. Although several traditional approaches based on morphological, biochemical, and environmental indicators have been proposed, their accuracy remains limited and strongly influenced by multiple postmortem and environmental variables [3–5, 75].

The present systematic review identified 64 studies investigating AI-based approaches for PMI estimation across multiple forensic domains. Overall, the results demonstrate a rapidly growing interest in the application of machine learning models to decomposition-related data, reflecting the increasing availability of high-throughput biological datasets and advanced computational methods.

Among the included studies, microbiome-based models represented the most frequently investigated approach, accounting for nearly half of the included studies. Microbial succession during decomposition has long been recognized as a potentially informative indicator of postmortem changes, and recent advances in sequencing technologies have enabled detailed characterization of microbial communities in different anatomical sites and environmental contexts.

Several studies applied machine learning algorithms to microbial community datasets obtained from the skin, gut, oral cavity, bone, and surrounding soil environments [13, 28, 29, 62]. Random Forest models were particularly frequently used for PMI prediction due to their robustness in handling complex nonlinear relationships and high-dimensional datasets [18, 43, 51]. In several investigations, microbiome-based models achieved high predictive performance, with coefficients of determination approaching R^2^ ≈ 0.95 or mean absolute errors of only a few hours to several days depending on the decomposition stage [43, 51].

Despite these promising results, microbiome-based approaches also present important limitations. Microbial community composition is strongly influenced by environmental factors such as temperature, humidity, and soil characteristics, which may limit the generalizability of predictive models across geographic regions. Furthermore, many studies were conducted using controlled animal decomposition models, including rodents or pigs, rather than human forensic cases [23, 56, 62].

Overall, predictive performance appeared to vary according to both the biological matrix analyzed and the investigated PMI interval. Microbiome-based models generally demonstrated the most stable predictive performance across broad decomposition windows, likely because microbial succession continues throughout advanced decomposition stages. In contrast, metabolomics and spectroscopy-based approaches appeared particularly effective during early PMI intervals, when biochemical alterations occur rapidly after death.

Imaging-based artificial intelligence models also demonstrated promising performance for early PMI estimation, especially in controlled experimental settings and postmortem imaging environments. However, their performance may be more sensitive to imaging standardization and acquisition protocols.

Several studies suggested that multimodal approaches integrating multiple biological and environmental datasets may achieve superior predictive performance compared with single-modality models, although direct comparative evidence remains limited.

Another important group of studies explored molecular approaches, including metabolomics and proteomics. These methods aim to identify biochemical signatures associated with postmortem degradation processes, such as protein fragmentation or metabolite accumulation.

Metabolomic profiling of tissues or biological fluids combined with machine learning algorithms has demonstrated promising results for early PMI estimation [25, 38, 47]. In particular, large-scale metabolomic analyses using neural networks or ensemble learning approaches have reported predictive errors of only a few days in human forensic datasets [47].

Proteomic studies have also shown encouraging results, particularly when analyzing skeletal muscle or bone protein degradation patterns [24, 27, 71]. In some cases, classification models based on proteomic features achieved very high accuracy for distinguishing PMI intervals.

Nevertheless, the application of molecular techniques in routine forensic practice remains limited by the need for specialized analytical equipment, such as mass spectrometry platforms, as well as the potential influence of ante-mortem physiological factors or environmental variables on molecular biomarkers.

Recent studies have increasingly investigated the use of imaging-based artificial intelligence models for PMI estimation. These approaches exploit high-dimensional datasets derived from postmortem imaging modalities such as computed tomography, histopathology slides, or hyperspectral imaging. Deep learning architectures including convolutional neural networks and Vision Transformer models have been applied to detect subtle structural or morphological changes associated with decomposition processes [30, 41, 64]. Similarly, machine learning analysis of postmortem CT radiomic features has been proposed as a tool for distinguishing early PMI intervals [33].

Although these approaches remain relatively underrepresented compared with microbiome-based methods, imaging-based AI models may provide valuable tools for early postmortem interval estimation, particularly in hospital or forensic imaging settings where postmortem CT is increasingly used.

Spectroscopy-based approaches represent another emerging research direction in AI-based PMI estimation. Techniques such as Fourier transform infrared spectroscopy (FTIR), attenuated total reflectance FTIR, and near-infrared spectroscopy have been used to detect biochemical changes in tissues or body fluids during decomposition [22, 65, 66].

Machine learning algorithms applied to spectroscopic datasets have demonstrated promising predictive performance, particularly for early PMI intervals where biochemical changes occur rapidly.

Similarly, several studies have applied machine learning models to forensic entomology datasets, including insect developmental stages or chemical signatures in puparia [15, 54, 69]. Because insect colonization often represents the most reliable indicator for longer postmortem intervals, AI-based entomological models may play a key role in extending PMI estimation beyond the early decomposition stages.

Although several studies reported highly promising predictive performance, direct comparisons between AI-based approaches and traditional PMI estimation methods were relatively limited. In many investigations, AI models were evaluated primarily within experimental datasets without systematic comparison against conventional forensic techniques such as body cooling curves, rigor mortis, or entomological staging performed by standard methodologies.

Consequently, while AI-based approaches appear capable of improving prediction accuracy in multidimensional datasets, current evidence should still be interpreted cautiously until larger externally validated comparative forensic studies become available.

Several limitations of this systematic review should be acknowledged. First, the literature search was limited to two major scientific databases (PubMed/MEDLINE and Scopus), and although additional citation tracking was performed, relevant studies indexed in other databases may have been missed. Second, only studies published in English were included, which may have introduced language bias. Another important limitation concerns the substantial heterogeneity across the included studies. Differences in data modalities, biological samples, decomposition conditions, AI model architectures, validation strategies, and reported performance metrics made direct comparison between studies challenging.

An important methodological limitation emerging from this review is the relatively limited use of independent external validation cohorts. Most studies relied on internal validation strategies, such as train–test splits or cross-validation, which may overestimate predictive performance and reduce generalizability in real forensic contexts.Furthermore, many of the included studies were based on experimental animal models rather than human forensic cases, which may limit the generalizability of the findings to real-world forensic investigations. Finally, because of the variability in study design and outcome reporting, it was not possible to perform a quantitative meta-analysis of model performance. Instead, results were synthesized narratively with a focus on methodological trends, data sources, and reported predictive performance. The methodological quality assessment further highlighted that many currently available AI-based PMI studies remain exploratory in nature, with limited external validation and reduced generalizability to real forensic scenarios.

In addition to methodological aspects, the increasing use of AI in forensic and medical contexts also raises relevant medico-legal considerations. AI-based models for PMI estimation may potentially contribute to forensic decision-making, making transparency in data processing and model validation particularly important. A recently proposed medico-legal framework for the evaluation of artificial intelligence in healthcare highlights the importance of systematically assessing dataset quality, identifying potential sources of error, and reconstructing causal pathways when algorithmic systems are involved in clinical or medico-legal contexts [76]. Although developed primarily for healthcare applications, similar principles may also be relevant for the evaluation and responsible use of AI-based tools in forensic investigations.

In addition, many machine learning approaches are inherently probabilistic and stochastic in nature, meaning that predicted PMI estimates are often associated with statistical uncertainty rather than deterministic conclusions. This aspect may have important implications in forensic and judicial contexts, where the interpretation, explainability, and reproducibility of AI-generated estimates become essential for evidentiary reliability and admissibility.

Future research should prioritize the development of large multicenter forensic datasets including diverse environmental conditions and human postmortem cases. Standardized data acquisition protocols and transparent reporting of machine learning pipelines would significantly improve reproducibility and comparability across studies.

In addition, the integration of multimodal datasets, combining microbiome profiles, molecular biomarkers, imaging features, and environmental variables, represents a promising direction for improving PMI prediction accuracy. Multimodal artificial intelligence models may be better suited to capture the complex biological processes involved in decomposition.

Finally, the development of interpretable AI models will be essential for forensic applications, where transparency and explainability are critical due to the potential legal implications of PMI estimation.

## Conclusions

Artificial intelligence has emerged as a powerful tool for improving the estimation of the postmortem interval by enabling the analysis of complex biological, molecular, and environmental datasets. The studies included in this systematic review demonstrate that AI-based approaches—particularly those based on microbial succession, molecular biomarkers, imaging data, and spectroscopic techniques—can achieve promising predictive performance across different PMI ranges.

Among the investigated strategies, microbiome-based models represent the most extensively explored approach, reflecting the strong association between microbial community dynamics and decomposition processes. At the same time, recent developments in metabolomics, proteomics, and deep learning–based imaging analysis highlight the growing diversification of data sources used for PMI prediction.

Despite these advances, several challenges remain. Many studies rely on small experimental datasets, limited external validation, and animal models that may not fully reproduce real forensic scenarios. In addition, the heterogeneity of analytical pipelines and performance metrics currently limits direct comparisons between studies.

Future research should therefore focus on the development of larger, standardized datasets derived from human forensic cases, the implementation of robust external validation strategies, and the integration of multimodal datasets combining biological, molecular, imaging, and environmental information. Such approaches may enable the development of more accurate, generalizable, and interpretable AI systems for forensic PMI estimation.

Overall, artificial intelligence represents a promising frontier in forensic science and has the potential to significantly improve the objectivity and reliability of postmortem interval estimation in future forensic investigations.

## Supplementary Information

Below is the link to the electronic supplementary material.

Supplementary Appendix 1: database search strategy.Supplementary Table S1: Risk-of-bias assessment of included studies according to adapted PROBAST/QUADAS-2 criteria for AI-based postmortem interval (PMI) prediction studies.Abbreviations: RoB – Risk of Bias.Supplementary Table S2: Summary of studies included in this systematic review investigating artificial intelligence (AI) approaches for postmortem interval (PMI) estimation. The table reports study characteristics, including first author and year of publication, data modality used for PMI prediction, biological sample type, sample size, artificial intelligence or machine learning model applied, validation strategy, target outcome (continuous PMI estimation or interval classification), and reported model performance metrics.Abbreviations: AI – Artificial Intelligence; ANN – Artificial Neural Network; AUC – Area Under the Receiver Operating Characteristic Curve; CNN – Convolutional Neural Network; FTIR – Fourier Transform Infrared Spectroscopy; GIS – Geographic Information System; MAE – Mean Absolute Error; ML – Machine Learning; NIR – Near-Infrared Spectroscopy; PMCT – Postmortem Computed Tomography; PMI – Postmortem Interval; PMImin – Minimum Postmortem Interval; PLS – Partial Least Squares; R² – Coefficient of Determination; RF – Random Forest; RMSE – Root Mean Square Error; SVR – Support Vector Regression; XGBoost – eXtreme Gradient Boosting.

## Data Availability

This study is a systematic review of previously published literature. No new datasets were generated during the current study. All data underlying the findings of this review are available in the published articles included in the review and in the supplementary materials provided by the respective authors.

## References

[1] Franceschetti L, Amadasi A, Bugelli V et al (2023) Estimation of late postmortem interval: where do we stand? A literature review. Biology (Basel) 12:783. 10.3390/biology12060783 37372068 10.3390/biology12060783 PMC10295266

[2] Wenzlow N, Mills D, Byrd J et al (2023) Review of the current and potential use of biological and molecular methods for the estimation of the postmortem interval in animals and humans. J Vet Diagn Invest 35:97–108. 10.1177/10406387231153930 36744749 10.1177/10406387231153930 PMC9999395

[3] Obafunwa JO, Roe A, Higley L (2025) A review of the estimation of postmortem interval using forensic entomology. Med Sci Law 65:52–64. 10.1177/00258024241275893 39285781 10.1177/00258024241275893

[4] Secco L, Palumbi S, Padalino P et al (2025) Omics” and postmortem interval estimation: a systematic review. Int J Mol Sci 26:1034. 10.3390/ijms26031034 39940802 10.3390/ijms26031034 PMC11817326

[5] Huang W, Zhao S, Liu H et al (2024) The role of protein degradation in estimation postmortem interval and confirmation of cause of death in forensic pathology: a literature review. Int J Mol Sci 25:1659. 10.3390/ijms25031659 38338938 10.3390/ijms25031659 PMC10855206

[6] Galante N, Cotroneo R, Furci D et al (2023) Applications of artificial intelligence in forensic sciences: current potential benefits, limitations and perspectives. Int J Legal Med 137:445–458. 10.1007/s00414-022-02928-5 36507961 10.1007/s00414-022-02928-5

[7] Sharma R, Diksha BAR et al (2022) Application of artificial intelligence and machine learning technology for the prediction of postmortem interval: A systematic review of preclinical and clinical studies. Forensic Sci Int 340:111473. 10.1016/j.forsciint.2022.111473 36166880 10.1016/j.forsciint.2022.111473

[8] AlJuhani AA, Desoky RM, Binshalhoub AA et al (2025) Advances in postmortem interval estimation: a systematic review of machine learning and metabolomics across various tissue types. Forensic Sci Med Pathol 21:1428–1446. 10.1007/s12024-025-01026-3 40702300 10.1007/s12024-025-01026-3

[9] Zar MS, Bibi A, Akhtar MS (2026) Unravelling time after death: a comprehensive review of multi-omics approaches in postmortem interval estimation. Forensic Sci Int 379:112779. 10.1016/j.forsciint.2025.112779 41411941 10.1016/j.forsciint.2025.112779

[10] Page MJ, McKenzie JE, Bossuyt PM et al (2021) The PRISMA 2020 statement: an updated guideline for reporting systematic reviews. BMJ 372:n71. 10.1136/bmj.n71 33782057 10.1136/bmj.n71 PMC8005924

[11] An G, Gao Y, Cheng S, Li N, Ren K, Du Q, Bai R, Sun J (2025) Artificial intelligence in forensic pathology: Multi-organ postmortem pathomics for estimating postmortem interval. Comput Methods Programs Biomed 270:108949. 10.1016/j.cmpb.2025.108949 40651440 10.1016/j.cmpb.2025.108949

[12] An G, Jing S, Cheng Z et al (2025) PMI estimation with cross-species transfer learning and visual information generated by pathomics foundation model. Int J Legal Med. 140, 1167–1182 10.1007/s00414-025-03659-z 10.1007/s00414-025-03659-z 41258447

[13] Belk A, Xu ZZ, Carter DO et al (2018) Microbiome data accurately predicts the postmortem interval using random forest regression models. Genes 9:104. 10.3390/genes9020104 29462950 10.3390/genes9020104 PMC5852600

[14] Bocaz-Beneventi G, Tagliaro F, Bortolotti F, Manetto G, Havel J (2002) Capillary zone electrophoresis and artificial neural networks for estimation of the post-mortem interval (PMI) using electrolytes measurements in human vitreous humour. Int J Legal Med 116(1):5–11. 10.1007/s004140100239 11924710 10.1007/s004140100239

[15] Butcher JB, Moore HE, Day CR, Adam CD, Drijfhout FP (2013) Artificial neural network analysis of hydrocarbon profiles for the ageing of Lucilia sericata for post mortem interval estimation. Forensic Sci Int 232(1–3):25–31. 10.1016/j.forsciint.2013.06.018 24053861 10.1016/j.forsciint.2013.06.018

[16] Cantürk İ, Özyılmaz L (2018) A computational approach to estimate postmortem interval using opacity development of eye for human subjects. Comput Biol Med 98:93–99. 10.1016/j.compbiomed.2018.04.023 29778926 10.1016/j.compbiomed.2018.04.023

[17] Cantürk İ, Özyılmaz L (2025) A deep feature driven expert system to estimate the postmortem interval from corneal opacity development. Expert Syst 42:e13757. 10.1111/exsy.13757 40423808 10.1007/s00414-025-03523-0

[18] Chen J, Wei Q, Yang F et al (2025) Unveiling the forensic potential of oral and nasal microbiota in post-mortem interval estimation. Int J Mol Sci 26:3432. 10.3390/ijms26073432 10.3390/ijms26073432 PMC11989810 40244278

[19] Chen S, Xia Y, Zhang X et al (2026) The impact of age and insects factors on cadaver microbial communities and application to postmortem interval estimation. Int J Legal Med 140:541-558. 10.1007/s00414-025-03621-z 10.1007/s00414-025-03621-z 41051498

[20] Cui C, Song Y, Mao D et al (2022) Predicting the postmortem interval based on gravesoil microbiome data and a random forest model. Microorganisms 11:56. 10.3390/microorganisms11010056 36677348 10.3390/microorganisms11010056 PMC9860995

[21] Deel H, Emmons AL, Kiely J et al (2021) A pilot study of microbial succession in human rib skeletal remains during terrestrial decomposition. mSphere 6:e00455-21. 10.1128/mSphere.00455-21 34259562 10.1128/mSphere.00455-21 PMC8386422

[22] Deng M, Wu H, Zhu Z, Wang Q, Liang X, Dong H, Guan L, Zhang J, Li B, Zhang M, Zhou H (2025) New perspective: An in vitro study on inferring the post-mortem interval (PMI) of human skeletal muscle based on ATR-FTIR spectroscopy combined with machine learning. Spectrochim Acta A Mol Biomol Spectrosc 339:126284. 10.1016/j.saa.2025.126284 40300229 10.1016/j.saa.2025.126284

[23] Finkelbergs D, Guo J, Huang Y et al (2022) Bacterial succession in microbial biofilm as a potential indicator for postmortem submersion interval estimation. Front Microbiol 13:951707. 10.3389/fmicb.2022.951707 35942315 10.3389/fmicb.2022.951707 PMC9356301

[24] Du QX, Zhang S, Long FH, Lu XJ, Wang Liang, Cao J, Jin QQ, Ren K, Zhang J, Huang P, Sun JH (2023) Combining with lab-on-chip technology and multi-organ fusion strategy to estimate post-mortem interval of rat. Front Med 9:1083474. 10.3389/fmed.2022.1083474 36703889 10.3389/fmed.2022.1083474 PMC9871555

[25] Fan W, Dai X, Ye Y, Yang H, Sun Y, Wu J, Fu Y, Shi K, Chen X, Liao L (2025) Estimation of postmortem interval under different ambient temperatures based on multi-organ metabolomics and machine learning algorithm. Int J Legal Med 139(5):2561–2575. 10.1007/s00414-025-03523-0 40423808 10.1007/s00414-025-03523-0

[26] Forger LV, Woolf MS, Simmons TL et al (2019) A eukaryotic community succession based method for postmortem interval (PMI) estimation of decomposing porcine remains. Forensic Sci Int 302:109838. 10.1016/j.saa.2025.126284 31233889 10.1016/j.forsciint.2019.05.054

[27] Garcés-Parra C, Saldivia P, Hernández M et al (2024) Enhancing late postmortem interval prediction: A pilot study integrating proteomics and machine learning to distinguish human bone remains over 15 years. Biol Res 57:75. 10.1186/s40659-024-00552-8 39444040 10.1186/s40659-024-00552-8 PMC11515459

[28] Hu L, Xing Y, Jiang P et al (2021) Predicting the postmortem interval using human intestinal microbiome data and random forest algorithm. Sci Justice 61:516-527. 10.1016/j.scijus.2021.06.006 34482931 10.1016/j.scijus.2021.06.006

[29] Iancu L, Bonicelli A, Procopio N (2024) Decomposition in an extreme cold environment and associated microbiome-prediction model implications for the postmortem interval estimation. Front Microbiol 15:1392716. 10.3389/fmicb.2024.1392716 38803371 10.3389/fmicb.2024.1392716 PMC11128606

[30] Ivanov J, Ondruschka B, Heinemann A (2025) Predicting the postmortem interval using a 3D-convolutional neural network in postmortem computed tomography. Rechtsmedizin 35:213-218. 10.1007/s00194-025-00765-5 39103637 10.1007/s00414-024-03296-y

[31] Johnson HR, Trinidad DD, Guzman S, Khan Z, Parziale JV, DeBruyn JM, Lents NH (2016) A machine learning approach for using the postmortem skin microbiome to estimate the postmortem interval. PLoS One 11(12):e0167370. 10.1371/journal.pone.0167370 10.1371/journal.pone.0167370 PMC5179130 28005908

[32] Kaszubinski SF, Receveur JP, Wydra B, Smiles K, Wallace JR, Babcock NJ, Weatherbee CR, Benbow ME (2020) Cold case experiment demonstrates the potential utility of aquatic microbial community assembly in estimating a postmortem submersion interval. J Forensic Sci 65(4):1210–1220. 10.1111/1556-4029.14303 32073664 10.1111/1556-4029.14303

[33] Klontzas ME, Leventis D, Spanakis K, Karantanas AH, Michail K (2023) Post-mortem CT radiomics for the prediction of time since death. Eur Radiol 33(12):8387–8395. 10.3390/genes9020104 37329460 10.1007/s00330-023-09746-2

[34] Kõrgesaar K, Jordana X, Gallego G et al (2022) Taphonomic model of decomposition. Leg Med (Tokyo) 56:102031. 10.1016/j.legalmed.2022.102031 35123354 10.1016/j.legalmed.2022.102031

[35] Li C, Li Z, Tuo Y, Ma D, Shi Y, Zhang Q, Zhuo X, Deng K, Chen Y, Wang Z, Huang P (2017) MALDI-TOF MS as a novel tool for the estimation of postmortem interval in liver tissue samples. Sci Rep 7(1):4887. 10.1038/s41598-017-05216-0 28687792 10.1038/s41598-017-05216-0 PMC5501804

[36] Li J, Wu YJ, Liu MF et al (2024) Multi-omics integration strategy in the post mortem interval of forensic science. Talanta 268:125249. 10.1186/s13568-026-02023-7 37839320 10.1016/j.talanta.2023.125249

[37] Li W, Xing Y, Gan L et al (2023) Exploring the value of microorganisms in the appendix for inferring postmortem interval in Sprague Dawley rats using high-throughput sequencing. J Forensic Sci 68:163-175. 10.1111/1556-4029.15173 36440674 10.1111/1556-4029.15173

[38] Li N, Liang XR, Zhou SD, Wu Y, Lu XY, Zhang SR, Li J, Sun JH (2023) Exploring postmortem succession of rat intestinal microbiome for PMI based on machine learning algorithms and potential use for humans. Forensic Sci Int Genet 66:102904. 10.1016/j.fsigen.2023.102904 37307769 10.1016/j.fsigen.2023.102904

[39] Li R, Xu R, Liu Y, Zhang S, Yu J, Wang M, Han C, Qiao D (2025) A study of grading strategy on postmortem intervals (PMIs) estimation from hypothermia deaths based on non-targeted metabolomics and machine learning algorithms. Leg Med (Tokyo) 78:102687. 10.1016/j.legalmed.2025.102687 40829283 10.1016/j.legalmed.2025.102687

[40] Li J, Wu YJ, Lu XY, Zhang SR, Li N, Du QX, Cao J, Bai RF, Sun JH (2026) Semi-supervised learning with dynamic classifier selection for PMI estimation: An animal study. J Forensic Leg Med 117:103051. 10.1016/j.jflm.2025.103051 41406784 10.1016/j.jflm.2025.103051

[41] Liang X, Deng M, Zhu Z, Wang Q, Wu H, Liang X, Dong H, Guan L, Zhang J, Li B, Zhang M, Zhou H (2025) A novel approach for estimating postmortem intervals under varying temperature conditions using pathology images and artificial intelligence models. Int J Legal Med 139(4):1809–1819. 10.1007/s00414-025-03447-9 10.1007/s00414-025-03447-9 40019556

[42] Liang X, Wang G, Wang H, Zhu Z, Zhang W, Li Y, Luo J, Wu S, Chen R, Deng M, Wu H, Shen C, Hu G, Zhang K, Sun Q, Wang Z (2025) Artificial intelligence-assisted estimation of postmortem intervals in bacterially infected cadavers using pathological imaging across variable temperature conditions. Sci Justice 65(6):101344. 10.1016/j.scijus.2025.101344 41320460 10.1016/j.scijus.2025.101344

[43] Liu R, Gu Y, Shen M, Li H, Zhang K, Wang Q, Thomas T, Wang Z, Zhou H (2020) Predicting postmortem interval based on microbial community sequences and machine learning algorithms. Environ Microbiol 22(6):2273–2291. 10.1111/1462-2920.15000Liu 32227435 10.1111/1462-2920.15000

[44] Liu R, Wang Q, Zhang K, Wu H, Wang G, Cai L, Yu K, Sun Q, Fan S, Wang Z (2022) Analysis of postmortem intestinal microbiota successional patterns with application in postmortem interval estimation. Microb Ecol 84(4):1087–1102. 10.1007/s00248-021-01923-4 34775524 10.1007/s00248-021-01923-4

[45] Lu XJ, Li J, Wei X, Du QX, Wu YJ, Wang L, Jin QQ, Zhang J, Sun JH (2023) A novel method for determining postmortem interval based on the metabolomics of multiple organs combined with ensemble learning techniques. Int J Legal Med 137(1):237–249. 10.1007/s00414-022-02844-8 35661238 10.1007/s00414-022-02844-8

[46] Løber IM, Hedemann MS, Villesen P, Blaabjerg ME, Banner J, Dagnæs-Hansen F, Marcher CW, Stensballe A, Uhrenholt L (2025) Untangling the postmortem metabolome: A machine learning approach for accurate PMI estimation. Anal Chem 97(40):16123–16132. 10.1016/j.compbiomed.2018.04.023 40698418 10.1021/acs.analchem.4c05796

[47] Magnusson R, Söderberg C, Ward LJ, Arpe J, Kugelberg FC, Elmsjö A, Green H, Nyman E (2026) The human metabolome and machine learning improves predictions of the post-mortem interval. Nat Commun 17:1504. 10.1038/s41467-026-69158-w 10.1038/s41467-026-69158-w PMC12894911 41673021

[48] Mason AR, McKee-Zech HS, Steadman DW, Suttles CJ, Davenport KM, Campagna SR, DeBruyn JM (2024) Environmental predictors impact microbial-based postmortem interval (PMI) estimation models within human decomposition soils. PLoS One 19(10):e0311906. 10.1371/journal.pone.0311906 39392823 10.1371/journal.pone.0311906 PMC11469530

[49] Qu H, Zhang X, Ye C, Li Q, Guo Y, Gu L, Wang Y, Liang Q (2024) Combining spectrum and machine learning algorithms to predict the weathering time of empty puparia of Sarcophaga peregrina (Diptera: Sarcophagidae). Forensic Sci Int 361:112144. 10.1016/j.forsciint.2024.112144 39018983 10.1016/j.forsciint.2024.112144

[50] Randall S, Cartozzo C, Simmons T et al (2021) Prediction of minimum postmortem submersion interval (PMSI(min)) based on eukaryotic community succession onskeletal remains recovered from a lentic environment. Forensic Sci Int 323:110784. 10.1016/j.forsciint.2021.110784 33864992 10.1016/j.forsciint.2021.110784

[51] Ren L, Wang Z, Yang Z et al (2026) Microbial succession and machine learning for postmortem interval estimation in bromadiolone-poisoned cadavers. AMB Express. 10.1186/s13568-026-02023-7 41634480 10.1186/s13568-026-02023-7 PMC12960868

[52] Rose S, Johnson H, Cartozzo C, Swall JL, Simmons T, Singh B (2025) Testing the efficacy of surface swab sampling to determine postmortem submersion interval (PMSI), using the microbiome colonization of skeletal remains. J Forensic Sci 70(4):1261–1273. 10.1111/1556-4029.70039 40329496 10.1111/1556-4029.70039 PMC12223347

[53] Schmidt VM, Zelger P, Wöss C et al (2022) Post-mortem interval of human skeletal remains estimated with handheld NIR spectrometry. Biology (Basel) 11:1020. 10.3390/biology11071020 36101401 10.3390/biology11071020 PMC9312135

[54] Sharif S, Wunder C, Amendt J, Zehner R, Verhoff MA, Sguazzi G, Matuszewski S (2024) Variations in cuticular hydrocarbons of Calliphora vicina (Diptera: Calliphoridae) empty puparia: Insights for estimating late postmortem intervals. Int J Legal Med 138(6):2717–2733. 10.1007/s00414-024-03296-y 10.1007/s00414-024-03296-y 39103637

[55] Shen Z, Zhong Y, Wang Y et al (2024)A computational approach to estimate postmortem interval using postmortem computed tomography of multiple tissues based on animal experiments. Int J Legal Med 138:1093-1107. 10.1007/s00414-023-03127-6 37999765 10.1007/s00414-023-03127-6

[56] Su Q, Zhao X, Liao X, Han M, Pan H, Guan L, Wang Q, Wu H, Zhang J, Li B, Zhang M, Zhou H (2025) Microbial succession patterns for postmortem interval estimation in decomposed mouse cadavers: A comparative study of mechanical asphyxia and hemorrhagic shock. J Forensic Sci 70(5):1892–1907. 10.1111/1556-4029.70108 40524283 10.1111/1556-4029.70108

[57] Thümmel L, Amendt J (2026) Estimation of long term post mortem intervals by ATR-FTIR spectroscopy of weathered blow fly puparia. Spectrochim Acta A Mol Biomol Spectrosc 349:127363. 10.1016/j.saa.2025.127363 41422669 10.1016/j.saa.2025.127363

[58] Wang J, Liu Z, Ren J, Wang S, Han X, Jiao G, Wang X, Fu J, Yao L, Fu G, Han J (2024) A preliminary study characterizing temporal changes in soil bacterial communities after dismembered bones were buried. Electrophoresis 45(15-16):1370–1378. 10.1016/j.jflm.2025.103051 38332582 10.1002/elps.202300274

[59] Wang L, Zhang F, Zeng K, Zhou Y, Li X, Guo L, Liu Y, Liang X, Dong H, Guan L, Zhang J, Li B, Zhang M, Zhou H (2022) Microbial communities in the liver and brain are informative for postmortem submersion interval estimation in the late phase of decomposition: A study in mouse cadavers recovered from freshwater. Front Microbiol 13:1052808. 10.1007/s00248-021-01923-4 36458191 10.3389/fmicb.2022.1052808 PMC9705336

[60] Wang X, Le C, Jin X, Zhang J, Shi S, Liu Y, Jin S, Wang Z (2024) Estimating postmortem interval based on oral microbial community succession in rat cadavers. Heliyon 10(12):e31897. 10.1007/s00414-022-02844-8 38882314 10.1016/j.heliyon.2024.e31897 PMC11177140

[61] Weisensee KE, Tica CI, Atwell MM et al (2024) geoFOR: A collaborative forensic taphonomy database for estimating the postmortem interval. Forensic Sci Int 355:111934. 10.1021/acs.analchem.4c05796 38277912 10.1016/j.forsciint.2024.111934

[62] Yang T, Chen X, Xie Q et al (2025) Estimating postmortem interval of buried pig carcasses by integrating microbial succession patterns with machine learning algorithms. Microorganisms 14:6. 10.3390/microorganisms14010006 41597526 10.3390/microorganisms14010006 PMC12844239

[63] Yu D, Mai Y, Zhang L, Wang T, Guan L, Wu H, Wang Q, Zhang J, Li B, Zhang M, Zhou H (2025) Viral community succession during cadaver decomposition and its potential for estimating postmortem intervals. Appl Environ Microbiol 91(10):e01453-25. 10.1016/j.forsciint.2024.112144 40956075 10.1128/aem.01453-25 PMC12542767

[64] Yu G, Bai B, Zhou M et al (2026) Deep learning method based on image recognition for intrapuparial age and postmortem interval estimation in the forensically important Sarcophaga peregrina (Diptera: Sarcophagidae). Forensic Sci Int 379:112761. 10.1016/j.forsciint.2021.110784 41349266 10.1016/j.forsciint.2025.112761

[65] Zhang F, Wang P, Zeng K, Yuan H, Wang Z, Li X, Yuan H, Du S, Guan D, Wang L, Zhao R (2022) Postmortem submersion interval estimation of cadavers recovered from freshwater based on gut microbial community succession. Front Microbiol 13:988297. 10.3389/fmicb.2022.988297 36532467 10.3389/fmicb.2022.988297 PMC9756852

[66] Zhang J, Li B, Wang Q, Wei X, Feng W, Chen YJ, Huang P, Wang Z (2017) Application of Fourier transform infrared spectroscopy with chemometrics on postmortem interval estimation based on pericardial fluids. Sci Rep 7(1):18013. 10.1038/s41598-017-18228-7 29269843 10.1038/s41598-017-18228-7 PMC5740144

[67] Zhang J, Wei X, Huang J et al (2018) Attenuated total reflectance Fourier transform infrared (ATR-FTIR) spectral prediction of postmortem interval from vitreous humor samples. Anal Bioanal Chem 410:7611-7620. 10.1016/j.saa.2025.127363 30349991 10.1007/s00216-018-1367-1

[68] Zhang J, Wang M, Qi X et al (2021) Predicting the postmortem interval of burial cadavers based on microbial community succession. Forensic Sci Int Genet 52:102488. 10.1002/elps.202300274 33667880 10.1016/j.fsigen.2021.102488

[69] Zhang R, Gao Y, Hu G, Wang Y, Li L, Guo Y, Shao S, Liu S, Wang Y (2025) Age estimation of Phormia regina pupae based on ATR-FTIR and chemometrics. Spectrochim Acta A Mol Biomol Spectrosc 325:125175. 10.1016/j.saa.2024.125175 10.1016/j.saa.2024.125175 39306914

[70] Zhang X, Bai Y, Ngando FJ et al (2022) Predicting the weathering time by the empty puparium of Sarcophaga peregrina (Diptera: Sarcophagidae) with the ANN models. Insects 13:808. 10.1016/j.heliyon.2024.e31897 36135509 10.3390/insects13090808 PMC9502838

[71] Zhang X, Qu H, Zhou Z et al (2024) Age determination of Chrysomya megacephala pupae through reflectance and machine learning analysis. Insects 15:184. 10.1128/aem.01453-25 38535379 10.3390/insects15030184 PMC10970854

[72] Zhang XD, Jiang YR, Liang XR et al (2023) Postmortem interval estimation using protein chip technology combined with multivariate analysis methods. Fa Yi Xue Za Zhi 39:115-120. 10.12116/j.issn.1004-5619.2022.420407 37277373 10.12116/j.issn.1004-5619.2022.420407

[73] Zhang Y, Pechal JL, Schmidt CJ et al (2019) Machine learning performance in a microbial molecular autopsy context: A cross-sectional postmortem human population study. PLoS One 14:e0213829. 10.1016/j.fsigen.2021.102488 30986212 10.1371/journal.pone.0213829 PMC6464165

[74] Zhao X, Zhong Z, Hua Z (2022) Estimation of the post-mortem interval by modelling the changes in oral bacterial diversity during decomposition. J Appl Microbiol 133(6):3451–3464. 10.1111/jam.15771 35950442 10.1111/jam.15771 PMC9825971

[75] Cecchi R, Camatti J, Schirripa ML et al (2025) Postmortem biochemistry of GFAP, NSE and S100B in cerebrospinal fluid and in vitreous humor for estimation of postmortem interval: A pilot study. Forensic Sci Med Pathol 21:589-598. 10.1007/s12024-024-00874-9 39147943 10.1007/s12024-024-00874-9

[76] Cecchi R, Calabrò F, Camatti J et al (2026) Artificial intelligence in healthcare: Proposal for a new medico-legal methodology in medical liability. Leg Med (Tokyo) 80:102764. 10.1371/journal.pone.0213829 41401747 10.1016/j.legalmed.2025.102764

