# Supplementary material for: Artificial intelligence in forensic science: a systematic review. Part II: long-range postmortem interval estimation
Source: Int J Legal Med. 2026 Jun 12;140(5):3331–43. doi: 10.1007/s00414-026-03856-4 (PMC13499855; doi:10.1007/s00414-026-03856-4)
Supplement: Supplementary file 1 [file 414_2026_3856_MOESM1_ESM.docx]

**Supplementary Appendix 1**

**Database search strategies**

**PubMed (MEDLINE)**

Search string:

(

("artificial intelligence" OR "machine learning" OR "deep learning"

OR "neural network*" OR "random forest" OR "support vector machine"

OR "predictive model*")

AND

("postmortem interval" OR "post-mortem interval"

OR "time since death" OR "time-of-death")

AND

(forensic OR "forensic pathology" OR decomposition OR cadaver)

)

**Scopus**

Search string:

TITLE-ABS-KEY(

("machine learning" OR "deep learning" OR "artificial intelligence"

OR "predictive model")

AND

("postmortem interval" OR "time since death")

AND

(forensic OR decomposition OR cadaver)

)

**Supplementary Table S1**

Risk-of-bias assessment of included studies according to adapted PROBAST/QUADAS-2 criteria for AI-based postmortem interval (PMI) prediction studies.

Abbreviations: RoB – Risk of Bias.

| **First Author, Year** | **Representativeness** | **Environmental Variability** | **Validation** | **Overfitting Risk** | **Reproducibility** | **Overall RoB** |
| --- | --- | --- | --- | --- | --- | --- |
| An 2025 | Moderate | Partial | External | Moderate | Moderate | Moderate |
| An 2026 | Moderate | Partial | Internal | Moderate | Moderate | Moderate |
| Belk 2018 | Moderate | Partial | Internal | Moderate | Moderate | Moderate |
| Bocaz-Beneventi 2002 | Moderate | Limited | Internal | High | Limited | High |
| Butcher 2013 | Limited | Limited | Internal | High | Limited | High |
| Cantürk 2018 | Limited | Limited | Internal | High | Limited | High |
| Cantürk 2025 | Moderate | Limited | LOSO validation | Moderate | Moderate | Moderate |
| Chen 2025 | Moderate | Partial | Internal | Moderate | Moderate | Moderate |
| Chen 2026 | Limited | Partial | Internal | Moderate | Moderate | Moderate |
| Cui 2022 | Limited | Partial | Internal | Moderate | Moderate | Moderate |
| Deel 2021 | Limited | Partial | Internal | Moderate | Moderate | Moderate |
| Deng 2025 | Moderate | Partial | Internal | Moderate | Moderate | Moderate |
| Finkelbergs 2022 | Moderate | Good | Internal | Moderate | Moderate | Moderate |
| Du 2023 | Moderate | Partial | External | Low | Moderate | Moderate |
| Fan 2025 | Limited | Good | Internal | Moderate | Moderate | Moderate |
| Forger 2019 | Limited | Partial | Internal | Moderate | Moderate | Moderate |
| Garcés-Parra 2024 | Moderate | Limited | Internal | Moderate | Moderate | Moderate |
| Hu 2021 | Moderate | Partial | Internal | Moderate | Moderate | Moderate |
| Iancu 2024 | Moderate | Good | Internal | Moderate | Moderate | Moderate |
| Ivanov 2025 | Moderate | Partial | Internal | Moderate | Moderate | Moderate |
| Johnson 2016 | Limited | Partial | Internal | High | Limited | High |
| Kaszubinski 2020 | Moderate | Good | Internal | Moderate | Moderate | Moderate |
| Klontzas 2023 | Moderate | Partial | Train/test split | Moderate | Moderate | Moderate |
| Korgesaar 2022 | Moderate | Good | Internal | Moderate | Moderate | Moderate |
| Li C. 2017 | Moderate | Partial | Cross-validation | Moderate | Moderate | Moderate |
| Li W. 2023 | Limited | Partial | Internal | Moderate | Moderate | Moderate |
| Li N. 2023 | Moderate | Partial | Internal | Moderate | Moderate | Moderate |
| Li J. 2024 | Moderate | Good | Internal | Moderate | Moderate | Moderate |
| Li R. 2025 | Moderate | Partial | Cross-validation | Moderate | Moderate | Moderate |
| Li J. 2026 | Moderate | Partial | Internal | Moderate | Moderate | Moderate |
| Liang 2025 | Moderate | Partial | External | Low | Moderate | Moderate |
| Liang 2025b | Moderate | Partial | External | Low | Moderate | Moderate |
| Liu 2020° | Moderate | Partial | Internal | Moderate | Moderate | Moderate |
| Liu 2020b | Moderate | Partial | Internal | Moderate | Moderate | Moderate |
| Lu 2022 | Moderate | Good | External | Low | Moderate | Moderate |
| Løber 2025 | Moderate | Partial | External | Low | High | Low |
| Magnusson 2026 | High | Good | External | Low | High | Low |
| Mason 2024 | Moderate | Good | Internal | Moderate | Moderate | Moderate |
| Qu 2024 | Moderate | Partial | Internal | Moderate | Moderate | Moderate |
| Randall 2021 | Moderate | Partial | Internal | Moderate | Moderate | Moderate |
| Reid 2023 | Moderate | Partial | Internal | Moderate | Moderate | Moderate |
| Ren 2025 | Moderate | Partial | Internal | Moderate | Moderate | Moderate |
| Rosato 2024 | Moderate | Partial | Internal | Moderate | Moderate | Moderate |
| Sampaio-Silva 2021 | Moderate | Good | Internal | Moderate | Moderate | Moderate |
| Shan 2023 | Moderate | Partial | Internal | Moderate | Moderate | Moderate |
| Shi 2025 | Moderate | Partial | Cross-validation | Moderate | Moderate | Moderate |
| Singh 2022 | Moderate | Partial | Internal | Moderate | Moderate | Moderate |
| Song 2024 | Moderate | Partial | Internal | Moderate | Moderate | Moderate |
| Sun 2025 | Moderate | Partial | Internal | Moderate | Moderate | Moderate |
| Tang 2023 | Moderate | Partial | Internal | Moderate | Moderate | Moderate |
| Tu 2025 | Moderate | Partial | External | Low | Moderate | Moderate |
| Ueland 2024 | Moderate | Good | External | Low | High | Low |
| Wang 2020 | Moderate | Partial | Internal | Moderate | Moderate | Moderate |
| Wang 2022 | Moderate | Partial | Cross-validation | Moderate | Moderate | Moderate |
| Wang 2024 | Moderate | Partial | Internal | Moderate | Moderate | Moderate |
| Wei 2025 | Moderate | Partial | Internal | Moderate | Moderate | Moderate |
| Wu 2021 | Moderate | Partial | Internal | Moderate | Moderate | Moderate |
| Xiao 2023 | Moderate | Partial | Internal | Moderate | Moderate | Moderate |
| Xu 2024 | Moderate | Partial | Internal | Moderate | Moderate | Moderate |
| Yang 2025 | Moderate | Good | Internal | Moderate | Moderate | Moderate |
| Yin 2023 | Moderate | Partial | Internal | Moderate | Moderate | Moderate |
| Yu 2022 | Moderate | Partial | Internal | Moderate | Moderate | Moderate |
| Zhang 2020 | Moderate | Partial | Internal | Moderate | Moderate | Moderate |
| Zhang 2023 | Moderate | Partial | Cross-validation | Moderate | Moderate | Moderate |
| Zhao 2025 | Moderate | Good | External | Low | High | Low |
| Zhou 2024 | Moderate | Partial | Internal | Moderate | Moderate | Moderate |
| Zhu 2025 | Moderate | Partial | Internal | Moderate | Moderate | Moderate |

Overall, most studies demonstrated moderate risk of bias due to limited external validation, experimental animal models, and relatively small datasets. Studies using human forensic datasets with independent validation cohorts generally showed lower methodological risk and greater generalizability.

Risk-of-bias assessment was adapted for AI-based forensic prediction studies and should be interpreted as a methodological appraisal rather than a formal clinical prediction-model assessment.

**Supplementary Table S2**

Summary of studies included in this systematic review investigating artificial intelligence (AI) approaches for postmortem interval (PMI) estimation. The table reports study characteristics, including first author and year of publication, data modality used for PMI prediction, biological sample type, sample size, artificial intelligence or machine learning model applied, validation strategy, target outcome (continuous PMI estimation or interval classification), and reported model performance metrics.

Abbreviations: AI – Artificial Intelligence; ANN – Artificial Neural Network; AUC – Area Under the Receiver Operating Characteristic Curve; CNN – Convolutional Neural Network; FTIR – Fourier Transform Infrared Spectroscopy; GIS – Geographic Information System; MAE – Mean Absolute Error; ML – Machine Learning; NIR – Near-Infrared Spectroscopy; PMCT – Postmortem Computed Tomography; PMI – Postmortem Interval; PMImin – Minimum Postmortem Interval; PLS – Partial Least Squares; R² – Coefficient of Determination; RF – Random Forest; RMSE – Root Mean Square Error; SVR – Support Vector Regression; XGBoost – eXtreme Gradient Boosting.

| **Ref** | **First Author** | **Year** | **Data Modality** | **Sample Type** | **Sample Size** | **AI / ML Model** | **Validation** | **Outcome Type** | **Performance Metrics** |
| --- | --- | --- | --- | --- | --- | --- | --- | --- | --- |
| 11 | An | 2025 | Histopathology imaging (pathomics) | Animal (Bama miniature pigs) | 12 pigs + 4 validation | DenseNet121, VGG16, stacking ensemble | External | PMI classification | Accuracy 93.75% internal, 87.5% external |
| 12 | An | 2026 | Histopathology imaging (WSI pathomics) | Animal + Human | 16 pigs + 23 humans | Vision Transformer (UNI), ResNet50, DenseNet121 | Internal | PMI classification | Accuracy 91.63% pigs, 78.95% human |
| 13 | Belk | 2018 | Microbiome sequencing | Soil and skin samples | Multiple datasets | Random Forest regression | Internal | Continuous PMI prediction | High predictive accuracy |
| 14 | Bocaz-Beneventi | 2002 | Biochemical (vitreous humour electrolytes) | Human | 61 cases | Artificial Neural Network | Internal | Continuous PMI | r² = 0.98 |
| 15 | Butcher | 2013 | Entomological chemistry (GC-MS hydrocarbons) | Lucilia sericata larvae | Experimental dataset | Artificial Neural Network | Internal | Age classification (PMI proxy) | Accuracy 80.8–87.7% |
| 16 | Cantürk | 2018 | Imaging (eye opacity) | Human | 10 subjects | Image processing + ML classifier | Internal | PMI prediction | Not reported |
| 17 | Cantürk | 2025 | Imaging (corneal opacity deep features) | Human | Not specified | AlexNet feature extraction + SVM/KNN | Leave-one-subject-out | PMI classification | Accuracy 0.96–0.97 |
| 18 | Chen | 2025 | Microbiome (oral and nasal cavities) | Human cadavers | 88 swab samples | Random Forest | Internal | Continuous PMI | MAE 2.16–5.14 days |
| 19 | Chen | 2026 | Microbiome (cadaver decomposition) | Rat cadavers | Experimental dataset | Random Forest | Internal | Continuous PMI | MAE 0.62–0.95 days |
| 20 | Cui | 2022 | Microbiome (gravesoil bacteria) | Mouse cadavers | Experimental dataset | Random Forest | Internal | Continuous PMI | MAE 1.27 days |
| 21 | Deel | 2021 | Microbiome (bone microbial succession) | Human skeletal remains | 6 human donors | Random Forest | Internal | Continuous PMI | Error ≈ ±34 days (1–9 months PMI) |
| 22 | Deng | 2025 | Spectroscopy (ATR-FTIR skeletal muscle) | Human skeletal muscle | Not specified | PLS-R, PCR, CLS-R, MLR, SVR, XGB-R | Internal | Continuous PMI | RMSE 1.8 days (MLR) |
| 23 | Finkelbergs | 2022 | Microbiome (epinecrotic biofilm in water) | Swine cadavers | Experimental model | Random Forest | Internal | Continuous PMI / PMSI | MAE ≈ 0.7–1.3 days |
| 24 | Du | 2023 | Proteomic fragments (lab-on-chip multi-organ) | Rat model | Experimental dataset | LR, SVM, RF, GBDT, MLPC + ensemble | External | PMI classification | Accuracy 0.962 internal / 0.893 external |
| 25 | Fan | 2025 | Metabolomics (GC-MS multi-organ) | Rat model | Experimental dataset | Support Vector Regression | Internal | Continuous PMI | High prediction accuracy across 5–35 °C |
| 26 | Forger | 2019 | Microbiome (eukaryotic succession) | Porcine model | 6 pigs | Random Forest | Internal | Continuous PMI | RMSE ≈ 6 days |
| 27 | Garcés-Parra | 2024 | Proteomics (bone proteins) | Human skeletal remains | Not specified | Random Forest | Internal | Late PMI classification | 100% accuracy for 15–20 years PMI |
| 28 | Hu | 2021 | Microbiome (intestinal microbiota) | Human cadavers | 63 cadavers / 87 samples | Random Forest | Internal | Continuous PMI | High predictive performance |
| 29 | Iancu | 2024 | Microbiome (cold-environment decomposition) | Animal model | 393 samples | Random Forest | Internal | Continuous PMI | MAE 1.36 weeks; R² = 0.91 |
| 30 | Ivanov | 2025 | Imaging (postmortem CT) | Human cadavers | 123 individuals / 544 PMCT scans | 3D-CNN (ResNet-18) | Internal | PMI classification | Best performance with 128×128 resolution |
| 31 | Johnson | 2016 | Skin microbiome (16S sequencing) | Human cadavers | Multiple cadaver samples | k-Nearest Neighbor regression | Internal | Continuous PMI | Error ±55 accumulated degree days |
| 32 | Kaszubinski | 2020 | Aquatic microbial community | Swine model | Experimental carcasses | Random Forest regression | Internal | PMSI estimation | Mean square error ≈ ±3 days |
| 33 | Klontzas | 2023 | PMCT radiomics | Human cadavers | 97 PMCT scans | XGBoost classifier | Internal (train/test split) | PMI classification (<12 h vs >12 h) | AUC 0.75 |
| 34 | Korgesaar | 2022 | Taphonomic variables + environmental factors | Human forensic cases | Retrospective dataset | Machine learning classifiers | Internal | PMI classification | Reliable classification of multiple PMI intervals |
| 35 | Li C. | 2017 | MALDI-TOF mass spectrometry (proteomic patterns) | Rat + human liver tissue | Experimental samples | PCA + neural network classifiers | Cross-validation | PMI classification | High recognition accuracy for PMI groups |
| 36 | Li W. | 2023 | Appendix microbiome sequencing | Rat model | Experimental dataset | Random Forest regression | Internal | Continuous PMI | MAE ≈10.27 h within 14 days |
| 37 | Li N. | 2023 | Rat intestinal microbiome | Rat model + human samples | Experimental dataset | Two-layer ML model (classification + regression) | Internal | Continuous PMI | Accuracy 90.48% for stage classification |
| 38 | Li J. | 2024 | Multi-omics (metabolomics + proteomics + FTIR) | Rat model | 112 rats | Stacking ensemble model | Internal | Continuous PMI | Accuracy 0.93, AUC 0.98, MAE 0.07 |
| 39 | Li R. | 2025 | Metabolomics (UPLC-MS/MS muscle) | Rat hypothermia model | 48 rats | KNN, SVM, RF, LR, DT, GNB | Cross-validation | PMI classification | High classification performance for multiple intervals |
| 40 | Li J. | 2026 | Proteomics (protein chip electrophoresis) | Rat skeletal muscle | Experimental dataset | Semi-supervised learning + dynamic classifier selection | Internal | Continuous PMI | AUC 0.89, R² 0.93 |
| 41 | Liang | 2025 | Pathology whole-slide imaging | Mouse cadavers | Experimental dataset | Deep learning (ResNet50 + ML classifiers) | External | PMI classification | Patch-level AUC ≥0.949; WSI-level AUC ≥0.800 |
| 42 | Liang | 2025 | Pathology imaging under infection conditions | Mouse cadavers | Experimental dataset | Deep learning + AI pathology analysis | External | PMI classification | Patch-level AUC ≥0.948 |
| 43 | Liu | 2020 | Intestinal microbiome (16S rRNA) | Mouse model | 80 mice (multiple organs) | Random Forest regression | Internal | Continuous PMI | MAE ≈20.01 h; R² ≈0.95 |
| 44 | Liu | 2020 | Microbial community sequencing | Mouse model | Multiple organs (brain, heart, cecum) | ANN, RF, SVM | Internal | Continuous PMI | MAE ≈1.5 h within 24 h PMI |
| 45 | Lu | 2022 | Multi-organ metabolomics (UPLC-HRMS) | Rat model | 140 rats | Ensemble learning (stacking) | External | PMI classification | Accuracy ≈93%, AUC ≈0.96 |
| 46 | Løber | 2025 | Untargeted metabolomics (UHPLC-qTOF-MS) | Rat tissues | Experimental dataset | Random Forest + Lasso regression | Cross-validation + external | Continuous PMI | High predictive accuracy within first 4 days |
| 47 | Magnusson | 2026 | Human blood metabolome | Human forensic dataset | 4876 individuals + 512 validation | Neural network model | External | Continuous PMI | MAE ≈1.45 days (test set) |
| 48 | Mason | 2024 | Soil necrobiome + environmental variables | Human decomposition donors | 19 individuals | Random Forest regression | Internal | Continuous PMI | MAE ≈804–997 accumulated degree hours |
| 49 | Qu | 2024 | ATR-FTIR spectroscopy (puparia weathering) | Insect specimens | Multiple puparia samples | XGBoost regression | Internal | PMImin estimation | MAE ≈1.20 (best model) |
| 50 | Randall | 2021 | Aquatic eukaryotic community sequencing | Porcine skeletal remains | 200 bone samples | Random Forest regression | Internal | PMSImin estimation | Error ±63–104 days depending on bone type |
| 51 | Ren | 2026 | Gut and anus microbiome (16S rRNA sequencing) | Rat cadavers (poisoning model) | Experimental dataset | Gradient Boosting regression (GB), RF, SVR, LR | Internal | Continuous PMI | R² = 0.954; RMSE = 5.6 h |
| 52 | Rose | 2025 | Bone surface microbiome (aquatic decomposition) | Porcine skeletal remains | Longitudinal aquatic dataset | Random Forest regression | Internal | PMSI estimation | R² ≈ 0.83; RMSE ≈ 580–623 ADD |
| 53 | Schmidt | 2022 | NIR spectroscopy of bones | Human skeletal remains | 104 bone samples | Artificial Neural Network | Internal | PMI classification | Accuracy 90–100% across PMI classes |
| 54 | Sharif | 2024 | Cuticular hydrocarbons (GC-MS) from puparia | Blowfly puparia | Experimental dataset | XGBoost regression | Internal | PMImin estimation | Accurate prediction across environmental conditions |
| 55 | Shen | 2024 | Postmortem CT imaging (multi-tissue HU values) | Rabbit model | 10 rabbits | SVM, Random Forest, KNN + stacking model | Internal | PMI classification | Accuracy ≈93%; AUC ≈0.96 |
| 56 | Su | 2025 | Skin and gut microbiome (16S sequencing) | Mouse cadavers | Experimental dataset | Random Forest regression | Internal | Continuous PMI | MAE ≈2.15–2.52 days |
| 57 | Thümmel | 2026 | ATR-FTIR spectroscopy of puparia | Blowfly puparia | Experimental dataset | Support Vector Regression | Cross-validation | PMImin estimation | RMSE ≈1–1.5 months |
| 58 | Wang L. | 2022 | Liver and brain microbiome (16S rDNA) | Mouse freshwater immersion model | Experimental dataset | Random Forest | Internal | PMSI estimation | MAE ≈0.99–1.28 days |
| 59 | Wang X. | 2024 | Oral microbiome succession | Rat cadavers | 21 rats | Random Forest + Linear regression | Internal | Continuous PMI | Predictive microbial markers identified |
| 60 | Wang J. | 2024 | Burial soil microbiome | Porcine bones buried in soil | 10 femurs | Random Forest + Multiple linear regression | Internal | Burial time / PMI | MAE ≈55–56 ADD |
| 61 | Weisensee | 2024 | Forensic taphonomy database + environmental data (GIS + decomposition features) | Human decomposition cases | 2529 cases | Machine learning predictive model integrated with ArcGIS | Cross-validation | Continuous PMI | R² = 0.82 |
| 62 | Yang | 2026 | Microbial succession (16S rRNA sequencing) | Buried pig carcasses (skin + soil) | 6 pigs | Random Forest | Internal | Continuous PMI | MAE ≈4.9–5.6 days |
| 63 | Yu | 2025 | Viral metagenomics (virome succession) | Buried rat cadavers | Experimental dataset | Extremely Randomized Trees (ERT) | Train/test split | Continuous PMI | R² = 0.96; MAE = 2.54 days |
| 64 | Yu | 2026 | Intra-puparial morphology imaging | Blowfly pupae (Sarcophaga peregrina) | Experimental dataset | ResNet50 + Vision Transformer | Internal | PMImin estimation | Precision 94.0%, F1 ≈93.4% |
| 65 | Zhang J. | 2017 | FTIR spectroscopy of pericardial fluid | Rabbit cadavers | Experimental dataset | nu-SVM + PLS models | External | Continuous PMI | SVM model best predictive performance |
| 66 | Zhang J. | 2018 | FTIR spectroscopy of vitreous humor | Rabbit cadavers (0–48 h PMI) | 72 rabbits | ANN, SVR, Bayesian Ridge | 10-fold CV + validation | Continuous PMI | ANN R² = 0.983; error ≈2.0 h |
| 67 | Zhang Y. | 2019 | Human postmortem microbiome sequencing | Human death investigations | 188 cases | Random Forest, XGBoost, Neural networks | Internal | PMI classification / regression | XGBoost best predictive performance |
| 68 | Zhang J. | 2021 | Microbial succession in burial cadavers | Rat cadavers (skin, rectum, soil) | Experimental dataset | Random Forest | Internal | Continuous PMI | MAE ≈1.82–2.13 days |
| 69 | Zhang X. | 2022 | Cuticular hydrocarbons of empty puparium (GC-MS) | Sarcophaga peregrina puparia | Experimental dataset | ANN, SVR, PLS | Internal | Weathering time / PMI | ANN best predictive model |
| 70 | Zhang F. | 2022 | Gut microbiome succession in submerged corpses | Mouse freshwater decomposition model | Experimental dataset | Random Forest regression | Internal | PMSI estimation | MAE ≈0.82 ± 0.17 days |
| 71 | Zhang | 2023 | Proteomics (protein chip expression profiles) | Rat skeletal muscle + human skeletal muscle samples | 80 rat samples + 9 human samples | BP Neural Network + Fisher discriminant | Cross-validation + external | PMI classification | BP-NN accuracy 98.2% internal, 95.8% external |
| 72 | Zhang | 2024 | Hyperspectral imaging of blowfly pupae | *Chrysomya megacephala* pupae | Experimental dataset | RF, SVR (RBF), SVR (poly), XGBoost regression, Lasso | Train/test + 7-fold CV | Development time / PMImin | XGBR MAE ≈3.9–7.4 (best model) |
| 73 | Zhang | 2025 | ATR-FTIR spectroscopy + chemometrics | *Phormia regina* puparium | Experimental dataset | PLS-DA + Random Forest | Train/test validation | Development time / PMImin | PLS-DA accuracy 88%, RF 81% |
| 74 | Zhao | 2022 | Oral microbiome sequencing (16S rRNA) | Rat oral cavity during decomposition | 96 rats | Random Forest regression | Train/validation split | Continuous PMI | R² = 0.9394 |

**Supplementary Appendix 1**

**Database search strategies**

**PubMed (MEDLINE)**

Search string:

(

("artificial intelligence" OR "machine learning" OR "deep learning"

OR "neural network*" OR "random forest" OR "support vector machine"

OR "predictive model*")

AND

("postmortem interval" OR "post-mortem interval"

OR "time since death" OR "time-of-death")

AND

(forensic OR "forensic pathology" OR decomposition OR cadaver)

)

**Scopus**

Search string:

TITLE-ABS-KEY(

("machine learning" OR "deep learning" OR "artificial intelligence"

OR "predictive model")

AND

("postmortem interval" OR "time since death")

AND

(forensic OR decomposition OR cadaver)

)

**Supplementary Table S1**

Risk-of-bias assessment of included studies according to adapted PROBAST/QUADAS-2 criteria for AI-based postmortem interval (PMI) prediction studies.

Abbreviations: RoB – Risk of Bias.

| **First Author, Year** | **Representativeness** | **Environmental Variability** | **Validation** | **Overfitting Risk** | **Reproducibility** | **Overall RoB** |
| --- | --- | --- | --- | --- | --- | --- |
| An 2025 | Moderate | Partial | External | Moderate | Moderate | Moderate |
| An 2026 | Moderate | Partial | Internal | Moderate | Moderate | Moderate |
| Belk 2018 | Moderate | Partial | Internal | Moderate | Moderate | Moderate |
| Bocaz-Beneventi 2002 | Moderate | Limited | Internal | High | Limited | High |
| Butcher 2013 | Limited | Limited | Internal | High | Limited | High |
| Cantürk 2018 | Limited | Limited | Internal | High | Limited | High |
| Cantürk 2025 | Moderate | Limited | LOSO validation | Moderate | Moderate | Moderate |
| Chen 2025 | Moderate | Partial | Internal | Moderate | Moderate | Moderate |
| Chen 2026 | Limited | Partial | Internal | Moderate | Moderate | Moderate |
| Cui 2022 | Limited | Partial | Internal | Moderate | Moderate | Moderate |
| Deel 2021 | Limited | Partial | Internal | Moderate | Moderate | Moderate |
| Deng 2025 | Moderate | Partial | Internal | Moderate | Moderate | Moderate |
| Finkelbergs 2022 | Moderate | Good | Internal | Moderate | Moderate | Moderate |
| Du 2023 | Moderate | Partial | External | Low | Moderate | Moderate |
| Fan 2025 | Limited | Good | Internal | Moderate | Moderate | Moderate |
| Forger 2019 | Limited | Partial | Internal | Moderate | Moderate | Moderate |
| Garcés-Parra 2024 | Moderate | Limited | Internal | Moderate | Moderate | Moderate |
| Hu 2021 | Moderate | Partial | Internal | Moderate | Moderate | Moderate |
| Iancu 2024 | Moderate | Good | Internal | Moderate | Moderate | Moderate |
| Ivanov 2025 | Moderate | Partial | Internal | Moderate | Moderate | Moderate |
| Johnson 2016 | Limited | Partial | Internal | High | Limited | High |
| Kaszubinski 2020 | Moderate | Good | Internal | Moderate | Moderate | Moderate |
| Klontzas 2023 | Moderate | Partial | Train/test split | Moderate | Moderate | Moderate |
| Korgesaar 2022 | Moderate | Good | Internal | Moderate | Moderate | Moderate |
| Li C. 2017 | Moderate | Partial | Cross-validation | Moderate | Moderate | Moderate |
| Li W. 2023 | Limited | Partial | Internal | Moderate | Moderate | Moderate |
| Li N. 2023 | Moderate | Partial | Internal | Moderate | Moderate | Moderate |
| Li J. 2024 | Moderate | Good | Internal | Moderate | Moderate | Moderate |
| Li R. 2025 | Moderate | Partial | Cross-validation | Moderate | Moderate | Moderate |
| Li J. 2026 | Moderate | Partial | Internal | Moderate | Moderate | Moderate |
| Liang 2025 | Moderate | Partial | External | Low | Moderate | Moderate |
| Liang 2025b | Moderate | Partial | External | Low | Moderate | Moderate |
| Liu 2020° | Moderate | Partial | Internal | Moderate | Moderate | Moderate |
| Liu 2020b | Moderate | Partial | Internal | Moderate | Moderate | Moderate |
| Lu 2022 | Moderate | Good | External | Low | Moderate | Moderate |
| Løber 2025 | Moderate | Partial | External | Low | High | Low |
| Magnusson 2026 | High | Good | External | Low | High | Low |
| Mason 2024 | Moderate | Good | Internal | Moderate | Moderate | Moderate |
| Qu 2024 | Moderate | Partial | Internal | Moderate | Moderate | Moderate |
| Randall 2021 | Moderate | Partial | Internal | Moderate | Moderate | Moderate |
| Reid 2023 | Moderate | Partial | Internal | Moderate | Moderate | Moderate |
| Ren 2025 | Moderate | Partial | Internal | Moderate | Moderate | Moderate |
| Rosato 2024 | Moderate | Partial | Internal | Moderate | Moderate | Moderate |
| Sampaio-Silva 2021 | Moderate | Good | Internal | Moderate | Moderate | Moderate |
| Shan 2023 | Moderate | Partial | Internal | Moderate | Moderate | Moderate |
| Shi 2025 | Moderate | Partial | Cross-validation | Moderate | Moderate | Moderate |
| Singh 2022 | Moderate | Partial | Internal | Moderate | Moderate | Moderate |
| Song 2024 | Moderate | Partial | Internal | Moderate | Moderate | Moderate |
| Sun 2025 | Moderate | Partial | Internal | Moderate | Moderate | Moderate |
| Tang 2023 | Moderate | Partial | Internal | Moderate | Moderate | Moderate |
| Tu 2025 | Moderate | Partial | External | Low | Moderate | Moderate |
| Ueland 2024 | Moderate | Good | External | Low | High | Low |
| Wang 2020 | Moderate | Partial | Internal | Moderate | Moderate | Moderate |
| Wang 2022 | Moderate | Partial | Cross-validation | Moderate | Moderate | Moderate |
| Wang 2024 | Moderate | Partial | Internal | Moderate | Moderate | Moderate |
| Wei 2025 | Moderate | Partial | Internal | Moderate | Moderate | Moderate |
| Wu 2021 | Moderate | Partial | Internal | Moderate | Moderate | Moderate |
| Xiao 2023 | Moderate | Partial | Internal | Moderate | Moderate | Moderate |
| Xu 2024 | Moderate | Partial | Internal | Moderate | Moderate | Moderate |
| Yang 2025 | Moderate | Good | Internal | Moderate | Moderate | Moderate |
| Yin 2023 | Moderate | Partial | Internal | Moderate | Moderate | Moderate |
| Yu 2022 | Moderate | Partial | Internal | Moderate | Moderate | Moderate |
| Zhang 2020 | Moderate | Partial | Internal | Moderate | Moderate | Moderate |
| Zhang 2023 | Moderate | Partial | Cross-validation | Moderate | Moderate | Moderate |
| Zhao 2025 | Moderate | Good | External | Low | High | Low |
| Zhou 2024 | Moderate | Partial | Internal | Moderate | Moderate | Moderate |
| Zhu 2025 | Moderate | Partial | Internal | Moderate | Moderate | Moderate |

Overall, most studies demonstrated moderate risk of bias due to limited external validation, experimental animal models, and relatively small datasets. Studies using human forensic datasets with independent validation cohorts generally showed lower methodological risk and greater generalizability.

Risk-of-bias assessment was adapted for AI-based forensic prediction studies and should be interpreted as a methodological appraisal rather than a formal clinical prediction-model assessment.

**Supplementary Table S2**

Summary of studies included in this systematic review investigating artificial intelligence (AI) approaches for postmortem interval (PMI) estimation. The table reports study characteristics, including first author and year of publication, data modality used for PMI prediction, biological sample type, sample size, artificial intelligence or machine learning model applied, validation strategy, target outcome (continuous PMI estimation or interval classification), and reported model performance metrics.

Abbreviations: AI – Artificial Intelligence; ANN – Artificial Neural Network; AUC – Area Under the Receiver Operating Characteristic Curve; CNN – Convolutional Neural Network; FTIR – Fourier Transform Infrared Spectroscopy; GIS – Geographic Information System; MAE – Mean Absolute Error; ML – Machine Learning; NIR – Near-Infrared Spectroscopy; PMCT – Postmortem Computed Tomography; PMI – Postmortem Interval; PMImin – Minimum Postmortem Interval; PLS – Partial Least Squares; R² – Coefficient of Determination; RF – Random Forest; RMSE – Root Mean Square Error; SVR – Support Vector Regression; XGBoost – eXtreme Gradient Boosting.

| **Ref** | **First Author** | **Year** | **Data Modality** | **Sample Type** | **Sample Size** | **AI / ML Model** | **Validation** | **Outcome Type** | **Performance Metrics** |
| --- | --- | --- | --- | --- | --- | --- | --- | --- | --- |
| 11 | An | 2025 | Histopathology imaging (pathomics) | Animal (Bama miniature pigs) | 12 pigs + 4 validation | DenseNet121, VGG16, stacking ensemble | External | PMI classification | Accuracy 93.75% internal, 87.5% external |
| 12 | An | 2026 | Histopathology imaging (WSI pathomics) | Animal + Human | 16 pigs + 23 humans | Vision Transformer (UNI), ResNet50, DenseNet121 | Internal | PMI classification | Accuracy 91.63% pigs, 78.95% human |
| 13 | Belk | 2018 | Microbiome sequencing | Soil and skin samples | Multiple datasets | Random Forest regression | Internal | Continuous PMI prediction | High predictive accuracy |
| 14 | Bocaz-Beneventi | 2002 | Biochemical (vitreous humour electrolytes) | Human | 61 cases | Artificial Neural Network | Internal | Continuous PMI | r² = 0.98 |
| 15 | Butcher | 2013 | Entomological chemistry (GC-MS hydrocarbons) | Lucilia sericata larvae | Experimental dataset | Artificial Neural Network | Internal | Age classification (PMI proxy) | Accuracy 80.8–87.7% |
| 16 | Cantürk | 2018 | Imaging (eye opacity) | Human | 10 subjects | Image processing + ML classifier | Internal | PMI prediction | Not reported |
| 17 | Cantürk | 2025 | Imaging (corneal opacity deep features) | Human | Not specified | AlexNet feature extraction + SVM/KNN | Leave-one-subject-out | PMI classification | Accuracy 0.96–0.97 |
| 18 | Chen | 2025 | Microbiome (oral and nasal cavities) | Human cadavers | 88 swab samples | Random Forest | Internal | Continuous PMI | MAE 2.16–5.14 days |
| 19 | Chen | 2026 | Microbiome (cadaver decomposition) | Rat cadavers | Experimental dataset | Random Forest | Internal | Continuous PMI | MAE 0.62–0.95 days |
| 20 | Cui | 2022 | Microbiome (gravesoil bacteria) | Mouse cadavers | Experimental dataset | Random Forest | Internal | Continuous PMI | MAE 1.27 days |
| 21 | Deel | 2021 | Microbiome (bone microbial succession) | Human skeletal remains | 6 human donors | Random Forest | Internal | Continuous PMI | Error ≈ ±34 days (1–9 months PMI) |
| 22 | Deng | 2025 | Spectroscopy (ATR-FTIR skeletal muscle) | Human skeletal muscle | Not specified | PLS-R, PCR, CLS-R, MLR, SVR, XGB-R | Internal | Continuous PMI | RMSE 1.8 days (MLR) |
| 23 | Finkelbergs | 2022 | Microbiome (epinecrotic biofilm in water) | Swine cadavers | Experimental model | Random Forest | Internal | Continuous PMI / PMSI | MAE ≈ 0.7–1.3 days |
| 24 | Du | 2023 | Proteomic fragments (lab-on-chip multi-organ) | Rat model | Experimental dataset | LR, SVM, RF, GBDT, MLPC + ensemble | External | PMI classification | Accuracy 0.962 internal / 0.893 external |
| 25 | Fan | 2025 | Metabolomics (GC-MS multi-organ) | Rat model | Experimental dataset | Support Vector Regression | Internal | Continuous PMI | High prediction accuracy across 5–35 °C |
| 26 | Forger | 2019 | Microbiome (eukaryotic succession) | Porcine model | 6 pigs | Random Forest | Internal | Continuous PMI | RMSE ≈ 6 days |
| 27 | Garcés-Parra | 2024 | Proteomics (bone proteins) | Human skeletal remains | Not specified | Random Forest | Internal | Late PMI classification | 100% accuracy for 15–20 years PMI |
| 28 | Hu | 2021 | Microbiome (intestinal microbiota) | Human cadavers | 63 cadavers / 87 samples | Random Forest | Internal | Continuous PMI | High predictive performance |
| 29 | Iancu | 2024 | Microbiome (cold-environment decomposition) | Animal model | 393 samples | Random Forest | Internal | Continuous PMI | MAE 1.36 weeks; R² = 0.91 |
| 30 | Ivanov | 2025 | Imaging (postmortem CT) | Human cadavers | 123 individuals / 544 PMCT scans | 3D-CNN (ResNet-18) | Internal | PMI classification | Best performance with 128×128 resolution |
| 31 | Johnson | 2016 | Skin microbiome (16S sequencing) | Human cadavers | Multiple cadaver samples | k-Nearest Neighbor regression | Internal | Continuous PMI | Error ±55 accumulated degree days |
| 32 | Kaszubinski | 2020 | Aquatic microbial community | Swine model | Experimental carcasses | Random Forest regression | Internal | PMSI estimation | Mean square error ≈ ±3 days |
| 33 | Klontzas | 2023 | PMCT radiomics | Human cadavers | 97 PMCT scans | XGBoost classifier | Internal (train/test split) | PMI classification (<12 h vs >12 h) | AUC 0.75 |
| 34 | Korgesaar | 2022 | Taphonomic variables + environmental factors | Human forensic cases | Retrospective dataset | Machine learning classifiers | Internal | PMI classification | Reliable classification of multiple PMI intervals |
| 35 | Li C. | 2017 | MALDI-TOF mass spectrometry (proteomic patterns) | Rat + human liver tissue | Experimental samples | PCA + neural network classifiers | Cross-validation | PMI classification | High recognition accuracy for PMI groups |
| 36 | Li W. | 2023 | Appendix microbiome sequencing | Rat model | Experimental dataset | Random Forest regression | Internal | Continuous PMI | MAE ≈10.27 h within 14 days |
| 37 | Li N. | 2023 | Rat intestinal microbiome | Rat model + human samples | Experimental dataset | Two-layer ML model (classification + regression) | Internal | Continuous PMI | Accuracy 90.48% for stage classification |
| 38 | Li J. | 2024 | Multi-omics (metabolomics + proteomics + FTIR) | Rat model | 112 rats | Stacking ensemble model | Internal | Continuous PMI | Accuracy 0.93, AUC 0.98, MAE 0.07 |
| 39 | Li R. | 2025 | Metabolomics (UPLC-MS/MS muscle) | Rat hypothermia model | 48 rats | KNN, SVM, RF, LR, DT, GNB | Cross-validation | PMI classification | High classification performance for multiple intervals |
| 40 | Li J. | 2026 | Proteomics (protein chip electrophoresis) | Rat skeletal muscle | Experimental dataset | Semi-supervised learning + dynamic classifier selection | Internal | Continuous PMI | AUC 0.89, R² 0.93 |
| 41 | Liang | 2025 | Pathology whole-slide imaging | Mouse cadavers | Experimental dataset | Deep learning (ResNet50 + ML classifiers) | External | PMI classification | Patch-level AUC ≥0.949; WSI-level AUC ≥0.800 |
| 42 | Liang | 2025 | Pathology imaging under infection conditions | Mouse cadavers | Experimental dataset | Deep learning + AI pathology analysis | External | PMI classification | Patch-level AUC ≥0.948 |
| 43 | Liu | 2020 | Intestinal microbiome (16S rRNA) | Mouse model | 80 mice (multiple organs) | Random Forest regression | Internal | Continuous PMI | MAE ≈20.01 h; R² ≈0.95 |
| 44 | Liu | 2020 | Microbial community sequencing | Mouse model | Multiple organs (brain, heart, cecum) | ANN, RF, SVM | Internal | Continuous PMI | MAE ≈1.5 h within 24 h PMI |
| 45 | Lu | 2022 | Multi-organ metabolomics (UPLC-HRMS) | Rat model | 140 rats | Ensemble learning (stacking) | External | PMI classification | Accuracy ≈93%, AUC ≈0.96 |
| 46 | Løber | 2025 | Untargeted metabolomics (UHPLC-qTOF-MS) | Rat tissues | Experimental dataset | Random Forest + Lasso regression | Cross-validation + external | Continuous PMI | High predictive accuracy within first 4 days |
| 47 | Magnusson | 2026 | Human blood metabolome | Human forensic dataset | 4876 individuals + 512 validation | Neural network model | External | Continuous PMI | MAE ≈1.45 days (test set) |
| 48 | Mason | 2024 | Soil necrobiome + environmental variables | Human decomposition donors | 19 individuals | Random Forest regression | Internal | Continuous PMI | MAE ≈804–997 accumulated degree hours |
| 49 | Qu | 2024 | ATR-FTIR spectroscopy (puparia weathering) | Insect specimens | Multiple puparia samples | XGBoost regression | Internal | PMImin estimation | MAE ≈1.20 (best model) |
| 50 | Randall | 2021 | Aquatic eukaryotic community sequencing | Porcine skeletal remains | 200 bone samples | Random Forest regression | Internal | PMSImin estimation | Error ±63–104 days depending on bone type |
| 51 | Ren | 2026 | Gut and anus microbiome (16S rRNA sequencing) | Rat cadavers (poisoning model) | Experimental dataset | Gradient Boosting regression (GB), RF, SVR, LR | Internal | Continuous PMI | R² = 0.954; RMSE = 5.6 h |
| 52 | Rose | 2025 | Bone surface microbiome (aquatic decomposition) | Porcine skeletal remains | Longitudinal aquatic dataset | Random Forest regression | Internal | PMSI estimation | R² ≈ 0.83; RMSE ≈ 580–623 ADD |
| 53 | Schmidt | 2022 | NIR spectroscopy of bones | Human skeletal remains | 104 bone samples | Artificial Neural Network | Internal | PMI classification | Accuracy 90–100% across PMI classes |
| 54 | Sharif | 2024 | Cuticular hydrocarbons (GC-MS) from puparia | Blowfly puparia | Experimental dataset | XGBoost regression | Internal | PMImin estimation | Accurate prediction across environmental conditions |
| 55 | Shen | 2024 | Postmortem CT imaging (multi-tissue HU values) | Rabbit model | 10 rabbits | SVM, Random Forest, KNN + stacking model | Internal | PMI classification | Accuracy ≈93%; AUC ≈0.96 |
| 56 | Su | 2025 | Skin and gut microbiome (16S sequencing) | Mouse cadavers | Experimental dataset | Random Forest regression | Internal | Continuous PMI | MAE ≈2.15–2.52 days |
| 57 | Thümmel | 2026 | ATR-FTIR spectroscopy of puparia | Blowfly puparia | Experimental dataset | Support Vector Regression | Cross-validation | PMImin estimation | RMSE ≈1–1.5 months |
| 58 | Wang L. | 2022 | Liver and brain microbiome (16S rDNA) | Mouse freshwater immersion model | Experimental dataset | Random Forest | Internal | PMSI estimation | MAE ≈0.99–1.28 days |
| 59 | Wang X. | 2024 | Oral microbiome succession | Rat cadavers | 21 rats | Random Forest + Linear regression | Internal | Continuous PMI | Predictive microbial markers identified |
| 60 | Wang J. | 2024 | Burial soil microbiome | Porcine bones buried in soil | 10 femurs | Random Forest + Multiple linear regression | Internal | Burial time / PMI | MAE ≈55–56 ADD |
| 61 | Weisensee | 2024 | Forensic taphonomy database + environmental data (GIS + decomposition features) | Human decomposition cases | 2529 cases | Machine learning predictive model integrated with ArcGIS | Cross-validation | Continuous PMI | R² = 0.82 |
| 62 | Yang | 2026 | Microbial succession (16S rRNA sequencing) | Buried pig carcasses (skin + soil) | 6 pigs | Random Forest | Internal | Continuous PMI | MAE ≈4.9–5.6 days |
| 63 | Yu | 2025 | Viral metagenomics (virome succession) | Buried rat cadavers | Experimental dataset | Extremely Randomized Trees (ERT) | Train/test split | Continuous PMI | R² = 0.96; MAE = 2.54 days |
| 64 | Yu | 2026 | Intra-puparial morphology imaging | Blowfly pupae (Sarcophaga peregrina) | Experimental dataset | ResNet50 + Vision Transformer | Internal | PMImin estimation | Precision 94.0%, F1 ≈93.4% |
| 65 | Zhang J. | 2017 | FTIR spectroscopy of pericardial fluid | Rabbit cadavers | Experimental dataset | nu-SVM + PLS models | External | Continuous PMI | SVM model best predictive performance |
| 66 | Zhang J. | 2018 | FTIR spectroscopy of vitreous humor | Rabbit cadavers (0–48 h PMI) | 72 rabbits | ANN, SVR, Bayesian Ridge | 10-fold CV + validation | Continuous PMI | ANN R² = 0.983; error ≈2.0 h |
| 67 | Zhang Y. | 2019 | Human postmortem microbiome sequencing | Human death investigations | 188 cases | Random Forest, XGBoost, Neural networks | Internal | PMI classification / regression | XGBoost best predictive performance |
| 68 | Zhang J. | 2021 | Microbial succession in burial cadavers | Rat cadavers (skin, rectum, soil) | Experimental dataset | Random Forest | Internal | Continuous PMI | MAE ≈1.82–2.13 days |
| 69 | Zhang X. | 2022 | Cuticular hydrocarbons of empty puparium (GC-MS) | Sarcophaga peregrina puparia | Experimental dataset | ANN, SVR, PLS | Internal | Weathering time / PMI | ANN best predictive model |
| 70 | Zhang F. | 2022 | Gut microbiome succession in submerged corpses | Mouse freshwater decomposition model | Experimental dataset | Random Forest regression | Internal | PMSI estimation | MAE ≈0.82 ± 0.17 days |
| 71 | Zhang | 2023 | Proteomics (protein chip expression profiles) | Rat skeletal muscle + human skeletal muscle samples | 80 rat samples + 9 human samples | BP Neural Network + Fisher discriminant | Cross-validation + external | PMI classification | BP-NN accuracy 98.2% internal, 95.8% external |
| 72 | Zhang | 2024 | Hyperspectral imaging of blowfly pupae | *Chrysomya megacephala* pupae | Experimental dataset | RF, SVR (RBF), SVR (poly), XGBoost regression, Lasso | Train/test + 7-fold CV | Development time / PMImin | XGBR MAE ≈3.9–7.4 (best model) |
| 73 | Zhang | 2025 | ATR-FTIR spectroscopy + chemometrics | *Phormia regina* puparium | Experimental dataset | PLS-DA + Random Forest | Train/test validation | Development time / PMImin | PLS-DA accuracy 88%, RF 81% |
| 74 | Zhao | 2022 | Oral microbiome sequencing (16S rRNA) | Rat oral cavity during decomposition | 96 rats | Random Forest regression | Train/validation split | Continuous PMI | R² = 0.9394 |
